# Qualities of music-evoked autobiographical memories are associated with auditory features of the memory-evoking music

**DOI:** 10.1371/journal.pone.0329072

**Published:** 2025-08-20

**Authors:** Safiyyah Nawaz, Diana Omigie

**Affiliations:** Department of Psychology, Goldsmiths University of London, London, United Kingdom; University of Bergamo, ITALY

## Abstract

Studies of music-evoked autobiographical memories (MEAMs) show that music is a potent cue for retrieving vivid and self-relevant memories. However, whether and how musical features are able to predict the qualities of MEAMs – including their emotional qualities, phenomenological characteristics and retrieval efficiency – remains unclear. In our study, a sample of 233 adult participants identified a piece of music that evoked an autobiographical memory (AM) before providing a written description of the memory, and then evaluating its emotional and phenomenological content. Participants were then presented with excerpts of ten songs that were popular during their childhood and early adulthood and reported the same details for any AMs evoked. Features of all songs were extracted using the Spotify Web API and subjected to principal components analysis for dimension reduction. This revealed a primary auditory feature component – characterised by low energeticness and high acousticness – that was found to predict several qualities of the memory. Specifically, results showed that low energetic – high acoustic songs were associated with AMs characterised emotionally by aesthetic appreciation, adoration, calmness, romance and sadness, while high energetic – low acoustic songs were associated with AMs high in memory energeticness, amusement and excitement. Phenomenologically, AMs associated with low energetic – high acoustic songs were described as less social, and more vivid, unique and important, and, in terms of retrieval efficacy, tended to be retrieved more slowly. Our findings show for the first time the extent to which the qualities of MEAMs can be predicted by music’s stimulus features. Further, by taking into account how the AMs were evoked, and subjective factors related to the memory-evoking music such as liking and familiarity, our study provides insights into possible mechanisms underlying music-assisted memory encoding and retrieval. We discuss the implications of our findings for understanding the links between perception, emotion and memory processes, and make suggestions for future work that can advance this research area.

## Introduction

Decades of research have confirmed most people’s intuition of a compelling connection between music and autobiographical memory. Hearing a song and recalling a memory – complete with the sensations, feelings or thoughts that were experienced at the time – has been estimated to happen as often as daily [1] and as such studying music-evoked autobiographical memories presents a valuable opportunity to better understand complex memory retrieval processes.

Autobiographical memories (AMs) are complex recollections of episodes from individuals’ personal histories that include the representations of thoughts and emotions corresponding to those episodes. Interestingly, in addition to their high incidence, AMs show a large amount of variety. Amongst other distinctions, AMs can range from recollections of personally-significant periods of time to specific moments or events [2]; they can be activated voluntarily or involuntarily; and they can be cued by sensory stimuli or effortfully retrieved by direct search. Autobiographical reminiscence is held to serve a range of socio-cognitive functions that contribute to identity and self-development [3,4], and it is thus relevant for psychologists to seek a better understanding of AM retrieval and elaboration. Here we use music, a powerful cue for triggering vivid AMs [5] to explore a question of broad relevance; namely, the extent to which features of a memory cue can predict the qualities of retrieved AMs.

### Music as a retrieval cue for rich autobiographical memories

Music has long been shown to be important in shaping listeners’ identities and sense of self [6,7], motivating researchers to investigate how – and the extent to which – music may be uniquely able to evoke significant memories. To this end, studies of music-evoked autobiographical memories (MEAMs) have tended to compare AMs evoked by music to AMs evoked by other sensory-perceptual cues (such as images of famous faces, words, and food). Main conclusions from this research have been that music is a salient cue for retrieving vivid memories [5], and that MEAMs tend to be experienced as disproportionately positive (whereby even the memories cued by negative music are reported as more positive than those cued by other negative stimuli [8]).

Yet other research has examined whether differences between music cues, especially the emotions the music expresses, may be seen reflected in the nature of the AMs evoked by said music. This line of research, which has mostly used unfamiliar instrumental music as cues, has shown that the emotionality – specifically arousal and valence – of unfamiliar emotional music cues influences the nature of retrieved AMs. Specifically, AMs cued by positive music have been shown to be retrieved more quickly [9,10], more often [11], and to be reported as more positive [8] than those cued by negative music. Furthermore, memories cued by high arousing music have been reported as more positive and episodic, and as containing specific and contextual information about the memory [9].

Interestingly, while there is some evidence of an emotion-congruence effect, whereby the emotions reported as part of a memory tend to match the emotions expressed by the music cue [9], studies which have used arousal and valence to characterise the music and memories in question have often found interactions that are suggestive of a more complex relationship between music and memory emotionality (for instance, low valence/low arousal music cues being shown to lead to less positive memories than low valence/high arousal music [11,12]). Such findings raise the possibility that the emotionality of a memory may be influenced by features of the memory-evoking music stimuli other than its position in the 2-dimensional circumplex emotion model. Here, we suggest that in addition to factors such as music liking and familiarity, which have already been shown to predict aspects of MEAM retrieval and emotionality [12,13], other factors like the auditory features of the memory-evoking music are worthy of systematic exploration, with regard to the role that they may play in determining the qualities of memories that music induces.

### A data-driven musical-feature approach to MEAMs

Taken together, and especially given views that emotional experience may be more precisely represented by distinct emotion categories than by valence and arousal dimensions [14], previous studies that have characterised both music stimulus features and AM emotionality solely with regard to arousal and valence may be considered limited in fully exploring MEAM retrieval mechanisms. Put another way, as previous research has not included the breadth of distinct emotional experiences that can be reported or observed as a part of memories (e.g., romance or amusement), it remains unclear whether memories related to such states can be reliably triggered by manipulating the qualities of presented music.

Features of music like melody, harmony, rhythm, dynamics and timbre are known to influence music-induced and perceived emotions [15] but at present, the extent to which such musical features may also predict the nature of music-evoked memories remains unclear. While musico-acoustic features of music have been and can be explored in a range of different ways (often to explore how features of music predict key emotional responses to music), recent studies have leveraged Spotify’s Web API for its versatility and convenience. In one recent study, de Fleurian & Pearce used this tool to show that chills-evoking songs are less loud and energetic (relating to dynamic range, timbre, onset range and general entropy), have lower tempos, and are more acoustic and instrumental than matched non-chills songs [16]. Similarly, Baltazar & Västfjäll used the tool to demonstrate that, in addition to loudness and energy, features such as acousticness and instrumentalness may distinguish relaxing from non-relaxing pieces. [17].

To date, only one study has examined the relationship between musical features and autobiographical memories. In that study, Salakka and colleagues examined the acoustic profiles of familiar music that participants had rated for emotionality and autobiographical salience, and were able to identify timbral, tonal, and temporal features that predicted emotionality, familiarity and autobiographical salience of the memories [18]. Specifically, their results showed that songs with a weaker pulse, fewer higher-mid frequencies and less harmonics and high notes were associated with higher autobiographical salience. Interestingly, statistical analysis showed that the music’s emotional intensity mediated these effects, demonstrating that the experience of strong emotions may be the strongest predictor of autobiographical salience. Here, it is important to note that autobiographical memory in that study was explored with a sole focus on general autobiographical salience; that is by asking *‘how much personal memories did the song evoke?*’. Thus, with no further evaluations of AMs being made by participants, it remains unclear how features of music may influence the many other ways in which MEAMs are known to vary, from their level of vividness, uniqueness, and self-importance, to their associated emotions and the speed with which they are retrieved.

### The current study

From the studies discussed above, a picture emerges of music-evoked autobiographical memory as a complex phenomenon that can offer insight into several aspects of AM processes. The literature points towards music being a strong sensory cue that evokes memories through emotion-related processes, and which auditory features are likely to contribute to. While several studies have examined how arousal and valence, as well as music liking and familiarity, relate to characteristics of AM [9,10,12,13], and while one study has shown musical features to relate to autobiographical salience [18], it nevertheless remains to be systematically investigated how well auditory features of memory-evoking music can predict the nature of the memories they evoke.

In light of the gap in the literature, and in line with the approach taken by Jakubowski and Eerola [8], who examined effects on MEAMs of music and non-musical memory cues varying in arousal and valence, the current study therefore asked: 1) how music’s auditory features influence emotional qualities of recalled memories, 2) how music’s auditory features relate to the phenomenological characteristics of memories (that is, characteristics related to the content of the memory and its persistence in long-term AM), and finally, 3) how music’s auditory features influence the efficiency with which memories are retrieved.

To this end, features of memory-evoking songs were extracted using the Spotify API, and we asked how those features related to emotional qualities, phenomenological content of, and efficiency of retrieving AMs. Critically, we extended beyond dimensional (arousal-valence) approaches to characterising emotional qualities i) by asking participants to identify categorical emotions related to their memories, and ii) by analysing memory descriptions for inclusion of emotional and perceptual words. These analyses allowed us to investigate which categories of emotions are present in memories that music tends to evoke, and also allowed us to examine whether musical features have predictive power in which memories are evoked.

We conducted a principal components analysis (PCA), which we predicted would reveal two components of musical features more or less related to arousal and valence. In turn we anticipated that, in line with other work, arousal-related features might contribute to arousal, valence, vividness, uniqueness, and retrieval efficiency of AMs, while musical valence-related features would influence AMs’ valence, social content, importance, vividness, and retrieval efficiency. To pre-empt our results section, PCA revealed only one main component characterised by *both* arousal and valence-related music features and as such our evaluation of results is only broadly discussed in reference to our initial pre-registered predictions.

## Materials and methods

### Participants

In total, 233 adult participants completed the study, including 154 women, 69 men, 6 nonbinary individuals, and 4 preferring not to say with ages ranging from 18 to 76 years old (M = 37.65, SD = 16.15). The recruitment aim was 200 participants, as this exceeded sample sizes across six comparable studies (42–114 participants) that showed effects of music on AMs similar to those examined here [8–10]. Participants were recruited through online subject-recruiting platform Prolific [19], Goldsmiths University undergraduate participation scheme, word of mouth, and were also incentivized by the chance to win one of three £20 cash vouchers.

### Materials

#### Music stimuli.

In line with MEAM studies utilising a similar procedure [5,20–22], we used songs from the Billboard Hot 100 year-end charts between the years 1941 and 2022 to cue MEAMs. All musical stimuli were 15-second excerpts containing highly recognizable parts of the song, as shared and described by Belfi et al. [5].

Each participant was presented with 10 clips of songs that were on the Billboard Hot 100 charts during the years in which they were between the ages of 9 and 19, with one song selected for each year. We defined the ‘reminiscence bump’ as this age range (i.e., between ages 9 and 19 where 14 is the midpoint), as 14 has previously been identified as the peak age for music-related memories [23].

### Procedure

Participants completed the study using the online survey platform Qualtrics [24]. Participants first reported on one MEAM related to a self-selected song in the first part of the survey, then reported on experimenter selected music-cued MEAMs (as cued by songs from Billboard Hot 100) in the second part of the survey.

For self-selected AMs, participants were instructed to identify the title and artist of a song related to an AM as such:

*Think of a song that reliably evokes an autobiographical memory of yours. That is, when you think about or hear this song, you are taken back in time to a specific memory in which you can visualise where you are and what you’re doing from your own perspective.*

An autobiographical memory was defined as follows (as from [25])

*An* ***autobiographical memory*** *occurs when you remember personal experiences from your past. These memories may contain details about events, people, places, and time periods from your life. Such a memory could be of a unique event, such as a memory of your 10th birthday party, or a recurring event, such as a memory of walking your dog.’*

For the self-selected MEAM, participants were allowed to report any song of any genre or musical style as preferred. Participants were then prompted to write a description of the corresponding memory and provide responses to Likert questions related to emotionality (such as memory valence and arousal), phenomenological characteristics (such as vividness and uniqueness), and memory retrieval such as degree of spontaneity retrieving the memory (see Table 1 for full list of dependent variables). All Likert variables were intended to be reported on a 5-point scale for consistency; however, vividness was inadvertently rated on a 7-point scale. This discrepancy was identified after data collection had commenced, and as such, we retained the 7-point scale data for vividness in our analysis.

**Table 1:** Outcome variables list and measurement.

Variable	Outcome category	Measurement	Measured for self-selected or cued MEAMs
AM arousal	Emotionality	Likert rating: “How energizing did the experience reported in your memory feel at the time?”	Both
AM valence	Emotionality	Likert rating: “How negative or positive did the experience reported in your memory feel at the time?”	Both
Categorical Emotions	Emotionality	List of 34 emotions (as few or many able to be selected): admiration, adoration, aesthetic appreciation, amusement, anger, anxiety, awe, awkwardness, boredom, calmness, confusion, contempt, craving, disappointment, disgust, empathic pain, entrancement, envy, excitement, fear, guilt, horror, interest, joy, nostalgia, pride, relief, romance, sadness, satisfaction, sexual desire, surprise, sympathy, triumph	Both
Positive emotion words	Emotionality	LIWC analysis	Both
Negative emotion words	Emotionality	LIWC analysis	Both
AM vividness	Phenomenological characteristics	Likert rating: “How vivid is this memory in your mind?”	Both
AM uniqueness	Phenomenological characteristics	Likert rating: “How unique is the experience reported in your memory?”	Both
AM importance	Phenomenological characteristics	Likert rating: “How important is this memory to your life story?”	Both
AM social content	Phenomenological characteristics	Likert rating: “How social was the experience reported in your memory?”	Both
Age at time of memory	Phenomenological characteristics	Numerical	Both
Specificity of AM	Phenomenological characteristics	Forced-choice: 1. Memory for a specific event 2. Memory for a time period or time of your life 3. General memories (for example, memory of a person or a place)	Both
Music presence	Phenomenological characteristics	Yes/No/Not sure to the below question: *Was the piece of music that you just heard present during the experience reported in your memory? That is, did the memory involve a previous incident of listening to this same music?*	Both
‘Motion’ words	Phenomenological characteristics	LIWC Analysis	Both
‘Space’ words	Phenomenological characteristics	LIWC Analysis	Both
‘See’ words	Phenomenological characteristics	LIWC Analysis	Both
‘Hear’ words	Phenomenological characteristics	LIWC Analysis	Both
‘Feel’ words	Phenomenological characteristics	LIWC Analysis	Both
‘Social’ words	Phenomenological characteristics	LIWC Analysis	Both
Word count	Phenomenological characteristics	LIWC Analysis	Both
Retrieval speed	Retrieval efficiency	Calculated in time elapsed between onset of stimulus and participant indication of memory	Experimenter-cued
Spontaneity of AM	Retrieval efficiency	Likert rating	Experimenter-cued

Participants then selected all the categorical emotions they would use to describe the experience reported in the memory from a list of 34 emotions that were previously compiled by Cowen & Keltner, who in previous work derived the list from several emotion taxonomies to incorporate nuances of states varying in positive, negative and everyday emotions [14]. Finally, participants identified whether the piece of music was present during the recalled memory, a type of situation which we refer to as *music presence*. Questions about the memory were phrased to prompt participants to report regarding how the experience felt *at the time of it occurring*, rather than how they were feeling in the moment of recalling the memory. For instance, “How negative or positive did the experience reported in your memory feel at the time?” was asked rather than “how negative or positive was the memory?”, since the latter could erroneously prompt participants to evaluate the valence of the memory in the moment of recall.

For experimenter-cued MEAMs (after reporting their own AM as described above), participants were presented with ten songs from the Billboard Hot 100 charts randomly selected from their reminiscence bump years as described in Materials. In each trial, a song started playing automatically and participants were asked to indicate when they had recalled a memory by clicking a button on screen, but to refrain from clicking the button if no memory came to mind. If they indicated that they recalled a memory, participants were then asked to provide a description of the memory and to rate the same items as described for the self-selected AM; afterwards, they would be prompted to also rate their liking and the familiarity of the song. Retrieval speed was calculated as the duration of time between the start of the page and song presentation and when the participant indicated a memory. If no memory was recalled, they would not be shown the memory questions and would only be asked to rate their liking and familiarity of the song before moving to the next trial.

Before moving on to questionnaires, participants were given the opportunity, where they didn’t have the chance to report it in the original trial, to report on up to two memories that came to mind after the excerpt ended. In these cases, participants were asked to indicate the title and artist of the song, and provide the memory and ratings as described above. Nine participants provided an additional AM through this opportunity.

At the end, participants completed STOMP (Short Test of Musical Preferences; [26]), and VVIQ (Vividness of Visual Imagery Questionnaire; [27]) questionnaires and two questions assessing musicality. These data were the focus of other studies and so will not be included in the present study’s analysis.

### Ethics

The protocol of the current study received approval from the Goldsmiths Psychology Department Ethics Committee at Goldsmiths, University of London (approval number: PS130623SNS). Recruitment took place between July 8, 2023 and February 25, 2024, and written informed consent was obtained for all participants included in the study and documented via responses in the Qualtrics survey. Only participants over 18 years of age were permitted to participate.

### Analyses

#### Data Cleaning and Processing.

All statistical analyses were performed with R version 4.2.3. Prior to analysis, data were inspected to identify missing values, outliers, and to confirm that data met necessary assumptions for statistical models. Full documentation of the data cleaning process is included in Supplementary Information: S1 Appendix Data Cleaning and Assumptions.

#### Music features.

Auditory features of both experimenter-selected and self-selected songs were obtained using the R package *spotifyr*, which pulls track information from the Spotify Web API [28]. We identified nine auditory features of interest to include in our principal component analyses: acousticness, danceability, energy, instrumentalness, liveness, loudness, speechiness, tempo and valence [29]. Descriptions of the features can be seen in Spotify’s developer documentation and provided in Table 2.

**Table 2:** Spotify auditory features definitions.

Variable	Description
**acousticness**	A confidence measure from 0.0 to 1.0 of whether the track is acoustic. 1.0 represents high confidence the track is acoustic.
**danceability**	Danceability describes how suitable a track is for dancing based on a combination of musical elements including ***tempo, rhythm stability, beat strength, and overall regularity***. A value of 0.0 is least danceable and 1.0 is most danceable.
**energy**	Energy is a measure from 0.0 to 1.0 and represents a perceptual measure of intensity and activity. Typically, energetic tracks feel fast, loud, and noisy. For example, death metal has high energy, while a Bach prelude scores low on the scale. Perceptual features contributing to this attribute include ***dynamic range, perceived loudness, timbre, onset rate, and general entropy***.
**instrumentalness**	Predicts whether a track contains no vocals. “Ooh” and “aah” sounds are treated as instrumental in this context. Rap or spoken word tracks are clearly “vocal”. The closer the instrumentalness value is to 1.0, the greater likelihood the track contains no vocal content. Values above 0.5 are intended to represent instrumental tracks, but confidence is higher as the value approaches 1.0.
**liveness**	Detects the presence of an audience in the recording. Higher liveness values represent an increased probability that the track was performed live. A value above 0.8 provides strong likelihood that the track is live.
**loudness**	The overall loudness of a track in decibels (dB). Loudness values are averaged across the entire track and are useful for comparing relative loudness of tracks. Loudness is the quality of a sound that is **the primary psychological correlate of physical strength (amplitude)**. Values typically range between −60 and 0 db.
**speechiness**	Speechiness detects the presence of spoken words in a track. The more exclusively speech-like the recording (e.g., talk show, audio book, poetry), the closer to 1.0 the attribute value. Values above 0.66 describe tracks that are probably made entirely of spoken words. Values between 0.33 and 0.66 describe tracks that may contain both music and speech, either in sections or layered, including such cases as rap music. Values below 0.33 most likely represent music and other non-speech-like tracks.
**tempo**	The overall estimated tempo of a track in beats per minute (BPM). In musical terminology, tempo is the speed or pace of a given piece and derives directly from the **average beat duration**.
**valence**	A measure from 0.0 to 1.0 describing the musical positiveness conveyed by a track. Tracks with high valence sound more positive (e.g., happy, cheerful, euphoric), while tracks with low valence sound more negative (e.g., sad, depressed, angry).

#### Linguistic inquiry and word count (LIWC).

Descriptions of reported autobiographical memories were analysed using the Linguistic Inquiry and Word Count (LIWC) software, which processes excerpts of text and returns summary variables reflecting a composite usage of words belonging to specific categories (i.e., parts of speech, emotion content, perceptual words) [30]. We identified 8 categories to include in analyses: positive emotion and negative emotion (as measures of emotionality), and motion, space, see, hear, feel, and social (as measures of phenomenological characteristics, i.e., subjective qualities referring to the individual’s experience of the memory). Our selection was informed by previous work that has found MEAM descriptions (compared to descriptions of AMs cued by other stimuli) to be characterised by differences in these categories [21,22,25,31].

LIWC variables tend to be non-normally distributed with a high proportion of MEAMs having 0 values in LIWC categories. A new boolean dummy variable was therefore created for each of the eight above categories, with values coded either 0 or for 1, with scores of 0 remaining as 0, and above 0 being coded as a 1. For the LIWC word count variable, which did not have a high prevalence of 0 value entries but was still non-normally distributed, a median split was used to obtain 0 (low word count) and 1 (high word count) codes. Subsequently, logistic regression analyses were conducted to determine which music features increased likelihood of greater-than-zero/ higher scores in each of the LIWC categories.

#### Predicting characteristics of AMs.

In the interest of simplicity and parsimony, we favoured an analytic strategy that included only as many music feature dimensions as necessary to provide meaningful interpretation. Accordingly, a principal components analysis for reducing dimensionality was conducted on the nine auditory features obtained from the Spotify API (see Table 2). As this resulted in one component that described the majority of explained variance, our primary analyses examining emotionality, phenomenological characteristics, and retrieval efficiency (linear mixed effects models or mixed-effects logistic regressions models as appropriate, with individual participant and stimulus as random effects) therefore included this principal component as the sole music feature predictor (see Table 1). However, in the interest of comprehensiveness, we also provide an analogous secondary set of analyses where the nine auditory features, as well as two non-continuous variables of key and mode, are included as individual predictors.

For both types of models (using one main principle component feature or individual features as predictors), emotionality outcome variables comprised Likert ratings of valence and arousal of AM, occurrence of the most commonly reported categorical emotions and LIWC scores for positive and negative emotion words. Phenomenological characteristic outcome variables comprised Likert ratings of vividness, uniqueness, importance and social content, memory specificity, description word count, music presence (whether the song that evoked the memory was also present in the reported memory), age at time of memory and LIWC scores for perceptual and social word categories. Retrieval efficiency outcome variables comprised retrieval time and reported spontaneity of recall, and for experimenter-cued memories only, a logistic regression analysis was conducted to examine whether music features could predict the likelihood of the track evoking a memory.

#### Comparing memories in response to participant-selected and experimenter-selected stimuli.

Given the intrinsic differences between how they are cued, we sought to examine differences between the musical features of, as well as the memories evoked by, experimenter-cued versus participant-selected songs. To this end, we conducted two exploratory cluster analyses, one including measures of memory content (both emotional and phenomenological), and one including auditory features from Spotify in an attempt to see whether clusters relating to type of MEAM (self-selected or experimenter-cued) would form. However, no clusters were identified, so subsequent linear mixed models including memory type (experimenter vs self-selected) as a fixed effect were conducted for each of the measures originally included in cluster analyses. For all analyses, the level of significance was α = 0.05, except where noted as Bonferroni-corrected for multiple comparisons.

#### Exploratory analyses.

Finally, two further sets of exploratory analyses were conducted: one set to examine the predictive power of music features when music is not heard during retrieval (i.e., self-selected MEAMs), and one set to examine the predictive power of music features when other personal subjective information (e.g., music liking and familiarity) is available. In the former, mixed effects models as described in ‘Predicting characteristics of AMs’ section were carried out but only on self-selected MEAMs. In the latter, mixed effects models as described in ‘Predicting characteristics of AMs’ section were carried out but i) including music liking and familiarity in addition to the principal component retained from the PCA as predictors of memory outcomes and also ii) including only experimenter-cued MEAMs (as liking and familiarity data were not available for self-selected).

## Results

### Descriptive statistics

In total, 214 AMs were self-selected, and 1224 AMs were evoked from experimenter-selected tracks, for a total of 1438 MEAMs. On average, participants recalled 5.34 memories (SD = 2.71, range = 0–10) from 10 cued songs, meaning 53% of cues presented to participants successfully evoked memories. Descriptive statistics for Likert ratings of AM details are provided in Table 3.

**Table 3:** Descriptive statistics for AM Likert ratings.

Variable	Range	Mean rating	SD
Vividness	1 (not at all vivid) – 7 (extremely vivid)	4.74	1.71
Valence	1 (very negative) – 5 (very positive)	3.85	1.04
Arousal	1 (extremely calm) – 5 (extremely energising)	3.56	1.11
Uniqueness	1 (this kind of experience happens all the time) – 5 (this is a once in a lifetime experience)	2.75	1.26
Importance	1 (not at all important) – 5 (extremely important)	2.66	1.30
Social content	1 (not at all social) – 5 (extremely social)	3.15	1.41

### Principal components analysis of auditory features

**Fig 1:**
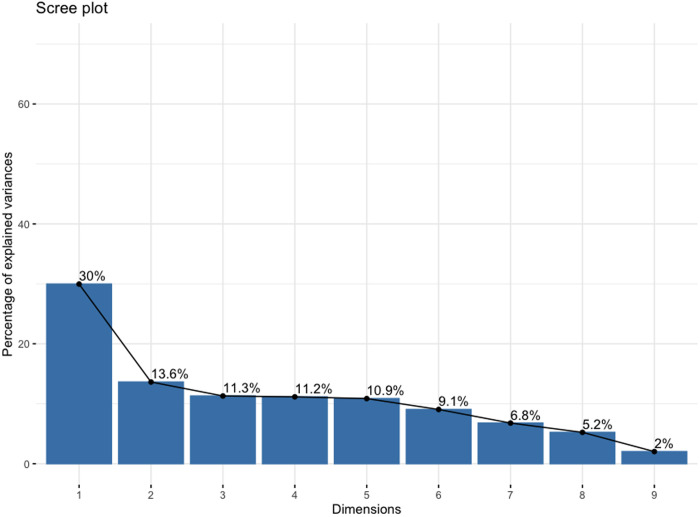
Scree plot of PCA components. Predictor variables included in PCA include nine auditory features: acousticness, danceability, energy, instrumentalness, liveness, loudness, speechiness, tempo and valence.

Our principal components analysis (PCA) of the auditory feature values (see Table 1) resulted in four components with an eigenvalue above 1. However, after inspection of the scree plot (see Fig 1), we retained one component that captured the majority of variance in the data and used this as a predictor in subsequent mixed effects models.

**Fig 2:**
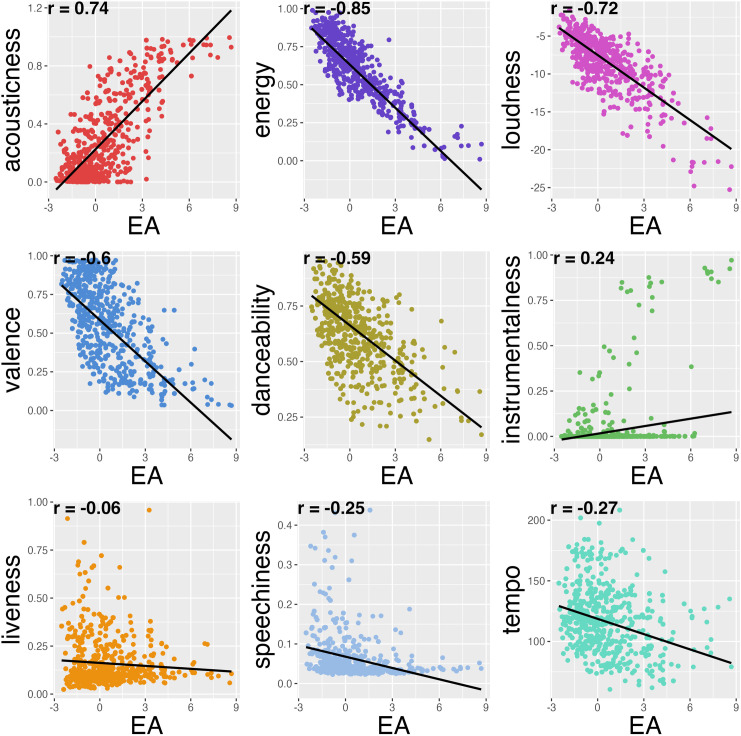
Scatterplots of E-A and each auditory feature. Correlation values are indicated in the top left corner of each scatterplot.

The one retained component had the highest positive loading from acousticness, and the highest negative loadings from energy and loudness, as well as moderate negative loadings from danceability and valence variables, (see Fig 2 for scatterplots and correlations between E-A and all included auditory features). We named this component energeticness-acousticness or E-A, based on the highest positive loading variable (acousticness) and highest negative loading variable (energy) to represent the auditory properties of individual songs as they range from high energy and low acoustic to low energy and high acoustic. Put simply, songs with high values in E-A are quieter, more acoustic, and have lower-energy perceptual features (ex: *re: stacks* – Bon Iver, *Clair de Lune* – Debussy) while songs with low values in E-A are louder, feel more energetic and are less acoustic (ex: *Trap Queen* – Fetty Wap, *When Doves Cry* – Prince). Linear mixed effects models to explore any relation E-A might have to liking and familiarity of songs showed it was neither related to liking (Estimate = −0.01, SE = 0.04, t(300.59) = −0.29, p = 0.77), nor familiarity (Estimate = −0.05, SE = 0.03, t(254.08) = −1.55, p = 0.12).

### Effect of E-A on emotional content of autobiographical memories

**Fig 3:**
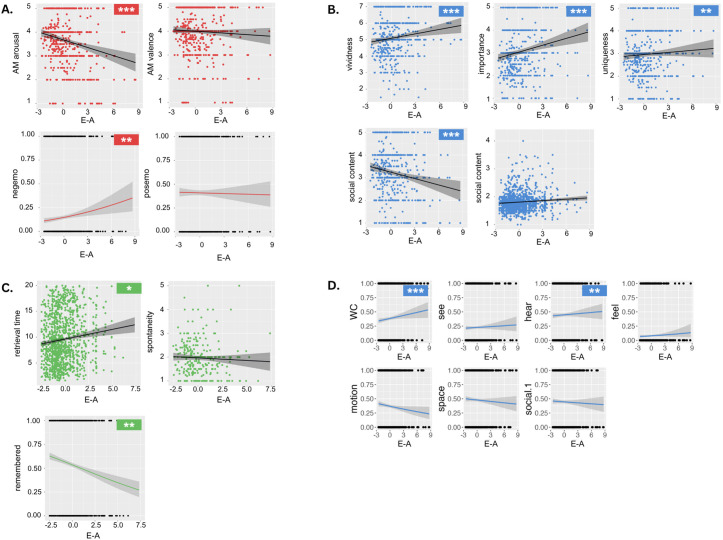
Plots showing E-A and outcome variables. A. Scatterplots and sigmoid function plots of E-A and MEAM emotional content outcomes B. Scatterplots of E-A and non-LIWC phenomenological characteristics. C. Scatterplots and sigmoid function plot of E-A and cue efficiency outcomes. D. Sigmoid function plots of E-A and LIWC phenomenological characteristic outcomes. Significance indicated as follows: *** p < 0.001; ** p < 0.01; * p < 0.05.

With regard to valence and arousal, E-A had no effect on AM valence or positive emotion words. However, it had a negative effect on AM arousal rating, (Estimate = −0.11, SE = 0.02, t(420.03) = −5.60, p < 0.001), and was associated with an increased likelihood of reporting LIWC negative emotion words (Estimate = 0.16, SE = 0.05, z = 3.32, p = 0.001). (see Fig 3 for plots, Table 4 for statistical reporting of all linear mixed effects models, Table 5 for statistical reporting of logistic mixed effects models).

**Table 4:** Estimate, standard error, t-value and p-values of linear mixed effects models of E-A for each outcome variable.

Dependent measure	*B*	SE	t	p
Emotionality				
1.Arousal***	−0.11	0.02	−5.60	< 0.001
2.Valence	−0.02	0.02	−1.01	0.31
Phenomenological characteristics				
1. Vividness***	0.10	0.03	3.45	0.001
2. Uniqueness**	0.06	0.02	2.75	0.006
3. Importance***	0.12	0.02	5.77	< 0.001
4. Social content***	−0.10	0.02	−4.02	< 0.001
5. Age at time of memory	0.09	0.10	0.89	0.38
Retrieval efficiency				
6. Retrieval time*	0.25	0.11	2.36	0.02
7. Spontaneity- deliberateness	−0.01	0.02	−0.34	0.73

Participant and song are included as random effects for each model.

**Table 5:** Estimate, standard error, z-value and p-values of mixed effects logistic regressions of E-A on reporting each outcome variable.

Dependent measure	*B*	SE	z	p
Emotionality reported For categorical emotions: * = significant at α = 0.0035 (0.05/14) ** = significant at α = 0.0007 (.01/14) *** = significant at α = 0.00007 (.001/14)				
1. LIWC – positive emotion	0.03	0.04	0.75	0.46
2. LIWC – negative emotion **	0.16	0.05	3.32	0.001
3. Admiration	0.08	0.06	1.36	0.18
4. Adoration***	0.24	0.06	3.98	< 0.001
5. Aesthetic appreciation*	0.21	0.07	2.94	0.003
6. Amusement***	−0.32	0.05	−6.46	< 0.001
7. Calmness***	0.45	0.06	7.20	< 0.001
8. Excitement***	−0.21	0.04	−4.96	< 0.001
9. Interest	−0.14	0.05	−2.84	0.005
10. Joy	−0.10	0.04	−2.54	0.011
11. Nostalgia	0.11	0.04	2.60	0.009
12. Satisfaction	−0.07	0.06	−1.18	0.24
13. Romance**	0.24	0.06	3.74	< 0.001
14. Awe	0.08	0.07	1.21	0.23
15. Sadness***	0.36	0.07	4.89	< 0.001
16. Awkwardness	−0.13	0.09	−1.51	0.13
Phenomenological characteristics				
1. Specificity	−0.02	0.04	−0.40	0.69
2. Music presence	0.00	0.04	0.06	0.95
3. Word count***	0.25	0.06	4.31	< 0.001
4. LIWC – seeing	0.08	0.04	1.96	0.05
5. LIWC – hearing**	0.14	0.04	3.20	0.001
6. LIWC – feeling	0.12	0.07	1.55	0.12
7. LIWC – motion	0.00	0.04	0.10	0.92
8. LIWC – space	0.03	0.05	0.64	0.52
9. LIWC – social	0.07	0.05	1.43	0.15
Retrieval efficiency				
10. Likelihood of evoking an AM**	−0.16	0.05	−2.97	0.003

Participant and song are included as random effects for each model.

To examine the predictive power of E-A with respect to AM-related categorical emotions, we first identified the most commonly reported emotion categories (by visual inspection of a bar plot of the number of times each emotion was reported, and including emotions associated with at least 100 individual AMs) and used logistic regression analysis to try to predict their occurrence with E-A. For these analyses, Bonferroni-corrections, with the corrected alpha level set at α = 0.05/ 14 = 0.0036, was applied due to multiple comparisons.

**Fig 4:**
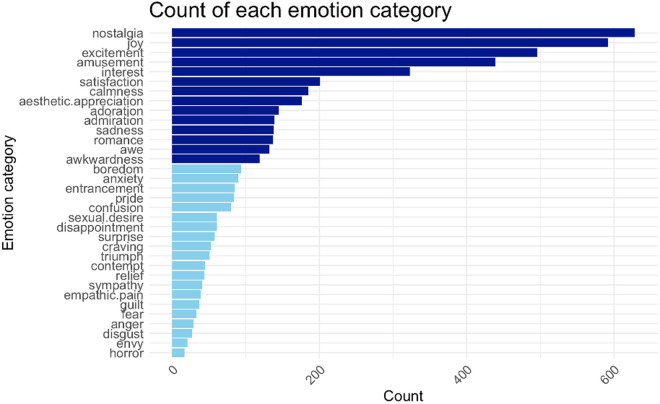
Occurrence of reported AM-associated categorical emotions. Values reflect sum of times each emotion was reported as associated with an AM. The top fourteen most commonly reported emotions used in analyses are indicated in dark blue.

**Fig 5:**
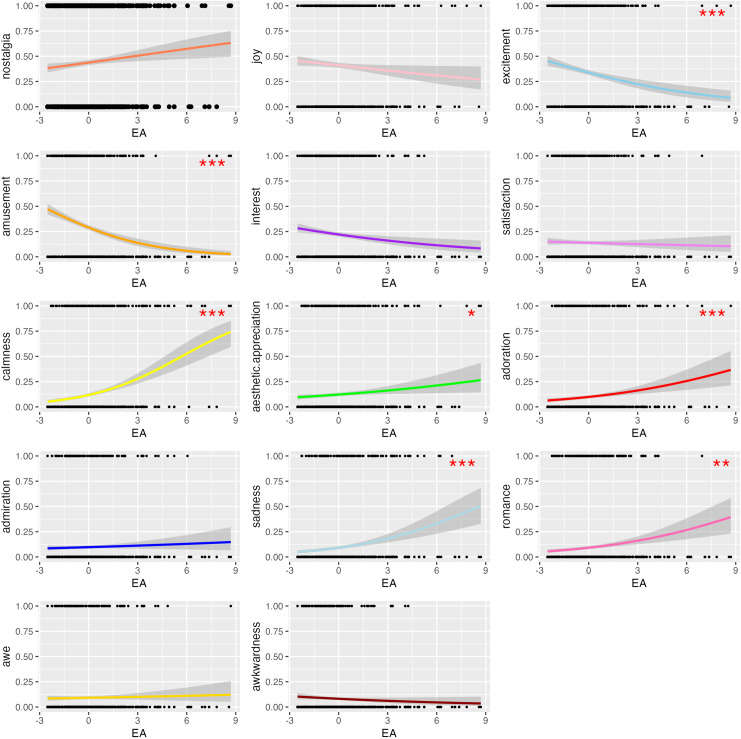
Sigmoid function plots of top emotion categories. Significant models are indicated with asterisks as follows: * = significant at α = 0.0035 (0.05/14); ** = significant at α = 0.0007 (.01/14); *** = significant at α = 0.00007 (.001/14).

Of the fourteen emotions included in analyses (see Fig 4 for occurrence of all emotion categories), E-A was not predictive of the occurrence of admiration, awe, awkwardness, interest, joy, nostalgia, or satisfaction. However, aesthetic appreciation (Estimate = 0.21, SE = 0.07, z = 2.94, p = 0.003), adoration (Estimate = 0.24, SE = 0.06, z = 3.98, p < 0.001), calmness (Estimate = 0.45, SE = 0.06, z = 7.20, p < 0.001), romance (Estimate = 0.24, SE = 0.06, z = 3.74, p < 0.001) and sadness (Estimate = 0.36, SE = 0.07, z = 4.89, p < 0.001) were predicted by increasing E-A while amusement (Estimate = −0.32, SE = 0.05, z = −6.46, p < 0.001) and excitement (Estimate = −0.21, SE = 0.04, z = −4.96, p < 0.001) were predicted by decreasing E-A (see Fig 5 for sigmoid function plots of most commonly reported emotions, Table 5 for statistical reporting of mixed effects logistic regressions).

### Effect of E-A on phenomenological content of autobiographical memories

Analysis examining the phenomenological properties of AMs showed a positive effect of E-A on memory vividness (Estimate = 0.10, SE = 0.03, t(504.98) = 3.45, p = 0.001), importance (Estimate = 0.12, SE = 0.02, t(337.10) = 5.77, p < 0.001) and uniqueness (Estimate = 0.06, SE = 0.02, t(401.48) = 2.75, p = 0.006), as well as a higher likelihood of high word count (Estimate = 0.21, SE = 0.05, z = 4.43, p < 0.001) and of reporting LIWC ‘hear’ words (Estimate = 0.14, SE = 0.04, z = 3.20, p = 0.001). Analyses also revealed a negative effect of E-A on social content (Estimate = −0.10, SE = 0.02, t(522.38) = −4.02, p < 0.001) (see Fig 3 for scatterplots, Tables 4 and 5 for statistical reporting). E-A had no relationship with memory specificity, age at the time of the memory reported, or music presence (whether the song that evoked the memory was present in the reported memory itself), nor with LIWC motion, space, visual, feeling or social words.

### Effect of E-A on retrieval efficiency of autobiographical memories

Examination of E-A’s effect on memory retrieval showed high E-A songs to be associated with longer memory retrieval time (Estimate = 0.25, SE = 0.11, t(332.30) = 2.36, p = 0.02), while logistic regression analysis showed that high E-A (experimenter-cued) songs were less likely to induce memories (Estimate = −0.16, SE = 0.05, z = −2.97, p = 0.003). No effect of E-A on spontaneity of memory was found. (See Fig 3 for plots, Tables 4 and 5 for statistical reporting).

### The effect of individual auditory features

To complement our primary analyses, and further assess the strength of individual auditory features as predictors of memory qualities, we replicated the above analyses with mixed-effects models that included the nine continuous auditory features used in the PCA and described in Table 1, as well as key (a categorical variable wherein integers map to pitch classes) and mode (a boolean variable representing major key as 1 and minor key as 0) as predictors.

**Fig 6:**
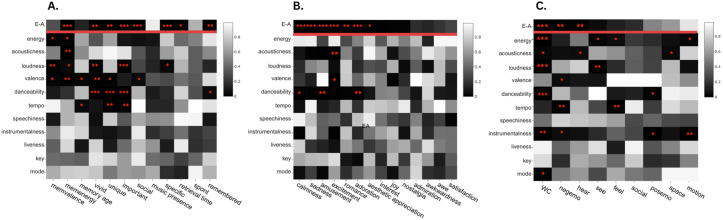
P-value heatmaps of mixed-effects models including individual auditory features as independent predictors of MEAM outcome variables. Note that E-A was not included as a predictor in these models but is added to these plots to allow the reader to compare the two analysis approaches. **A.** P-value heatmap for outcome variables excluding LIWC and categorical emotions. **B.** P-value heatmap for categorical emotions. **C.** P-value heatmap for LIWC categories. For plots A and C, * = significant at α = 0.05, ** = significant at α = 0.01, ** = significant at α = 0.001. For plot B, * = significant at α = 0.0035, ** = significant at α = 0.0007, ** = significant at α = 0.00007 reflecting Bonferroni corrections.

These analyses resulted in similar outcomes to models that used E-A as the single predictor: individual features predicted almost all of the same outcome variables as E-A for non-LIWC variables (as summarised in Tables 4 and 5), and the majority of independent features that significantly predicted outcomes loaded strongly onto E-A. For LIWC variables, some relationships were observed whereby individual features predicted the presence of certain LIWC categories that E-A did not predict (with energy predicting see, feel and motion words; acousticness predicting space words; loudness predicting see words; danceability predicting positive emotion words; tempo predicting feel words; and instrumentalness predicting positive emotion and motion words). We further point out that unlike in the E-A models, these sets of analyses showed i) higher instrumentalness, which did not strongly load onto E-A, to predict increased likelihood of MEAMs having high LIWC word count and including negative emotion, positive emotion and motion words, ii) lower tempo, which also did not load onto E-A, to predict high memory age, uniqueness, importance and presence of LIWC feel and negative emotion words, and finally iii) energy, loudness and valence to all independently predict memory valence (despite E-A not being able to predict memory valence). Results of these analyses are displayed in Fig 6 which shows p-values of mixed-effects models. All statistical output for these models is also reported in OSF at this link: https://osf.io/jke9w/

### Exploratory analyses

#### Comparing self-selected and experimenter-cued MEAMs.

Exploratory cluster analyses (as described in our pre-registration report) were conducted to explore patterns of reported autobiographical memories among participants in the study, and specifically to see whether any salient differences between self-selected and experimenter-cued MEAMs might be seen reflected in meaningful clusters. Hierarchical clustering with complete linkage and Euclidean distance as the similarity measure, however, failed to show distinct clusters.

Therefore mixed effects models were conducted for each variable that had been included in the cluster analysis, where MEAMs only corresponding to reminiscence bump age (between 9 and 19) were included to constitute a comparable sample. This was done as a majority of experimenter-cued MEAMs would have been from this time frame, and we aimed to identify differences on account of whether MEAMs were self-selected or cued, not on the basis of age of memory. To control for the increased risk of Type I errors due to multiple comparisons (n = 17), we applied Bonferroni corrections, with the corrected alpha level set at α = 0.05/ 17 = 0.0029.

Analyses revealed that self-selected memories were more specific (Estimate = 0.84, SE = 0.24, z = 3.53, p < 0.001), vivid (Estimate = 1.19, SE = 0.15, t(910.07) = 7.86, p < 0.001), positive (Estimate = 0.50, SE = 0.10, t(944.77) = 5.25), p < 0.001), arousing (Estimate = 0.43, SE = 0.10, t(851.70) = 4.12, p < 0.001), unique (Estimate = 0.74, SE = 0.10, t(848.98) = 7.20, p < 0.001), important (Estimate = 1.31, SE = 0.11, t(890.74) = 12.48, p < 0.001) and had a higher likelihood of having high word count (Estimate = 4.72, SE = 0.53, z = 8.95, p < 0.001). With regard to LIWC word ratings, self-selected memories also predicted a greater likelihood of positive emotion words (Estimate = 1.37, SE = 0.24, z = 5.71, p < 0.001), negative emotion words (Estimate = 1.12, SE = 0.24, z = 4.60, p < 0.001), motion words (Estimate = 2.11, SE = 0.29, z = 7.31, p < 0.001), space words (Estimate = 2.10, SE = 0.53, z = 3.95, p < 0.001), see words (Estimate = 1.10, SE = 0.24, z = 4.67, p < 0.001), hear words (Estimate = 1.79, SE = 0.00, z = 1614.89, p < 0.001), social words (Estimate = 2.25, SE = 0.53, z = 4.22, p < 0.001) and feeling words (Estimate = 7.28, SE = 0.00, z = 2712.79, p < 0.001). Analysis also revealed self-selected memories to be associated with higher values of E-A (Estimate = 0.37, SE = 0.03, t(768.79) = 13.43, p < 0.001).

#### Effects of E-A on self-selected MEAMs only.

Models examining the effect of E-A on self-selected MEAMs only were carried out to determine E-A’s predictive power in situations where listeners recount the memory without hearing the music. While this characteristic of the self-selected music cannot be isolated from the fact that self-selected MEAMs music i) is more personally salient (being self-selected) and ii) spans more varied genres and time periods than the experimenter selected billboard music, it is interesting to note what E-A continued to predict or not. Indeed, results showed that E-A continued to predict many aspects of emotionality, namely AM arousal (Estimate = −0.16, SE = 0.04, t(207.31) = −4.48, p < 0.001), likelihood of reporting negative words (Estimate = 0.19, SE = 0.07, z = 2.81, p = 0.005), calmness (Estimate = 0.26, SE = 0.07, z = 3.89, p < 0.001) and excitement (Estimate = −0.23, SE = 0.07, z = −3.15, p = 0.002); as well as the phenomenological quality of social content (Estimate = −0.14, SE = 0.04, t(212) = −3.41, p = 0.001.

Conversely, while we cannot rule out that this was due to the reduced sample size inevitable when looking at self-selected MEAMs only, these analyses showed E-A was not able to predict several categorical emotions (aesthetic appreciation, adoration, amusement, sadness or romance) and a number of key phenomenological variables (vividness, uniqueness, importance, high word count and likelihood of using ‘hear’ words).

#### Effects of E-A in models including music liking and familiarity.

Our results showed E-A to predict numerous aspects of MEAMs, even in the absence of the music. However, it remained unclear how well it would perform as a predictor of memory qualities when details of personal salience (i.e., liking and familiarity) of the music stimuli are also available. To this end, linear mixed effects models analogous to the above but including music liking and familiarity in addition to E-A, as predictors were run on experimenter-cued AMs. Results from these analyses showed E-A to continue to significantly predict several aspects of emotionality (arousal, reporting of calmness, amusement, excitement, and sadness), phenomenological characteristics (social content), and retrieval efficiency (likelihood of evoking memory and retrieval speed). However, we found that E-A no longer significantly predicted some aspects of emotionality: negative emotion words, likelihood of reporting certain emotions (aesthetic appreciation, adoration, and romance) and phenomenological characteristics: vividness, importance, uniqueness, likelihood of high word count and hearing words, and age at time of memory, with liking and/or familiarity shown to predict these outcome variables more strongly. In brief, these results showed that liking and familiarity, when available and included, may be more able to account for some effects initially attributed solely to E-A. Full results for these models are available in OSF: https://osf.io/jke9w/

## Discussion

This study aimed to examine how characteristics of music-evoked autobiographical memories may be influenced by auditory features of the same memory-evoking music in MEAMs that were either volunteered by participants or cued by experimenter-selected popular music. Analyses revealed a single musical feature component we named energeticness-acousticness or E-A. This component was shown to predict qualities related to the emotionality, phenomenological characteristics and retrieval efficiency of MEAMs, albeit only to a lesser extent in the presence of information about how much the music was familiar to or liked by the listener.

### Music features can predict categorical emotions associated with AMs

A large bulk of the research regarding MEAMs has characterised the emotionality of music and the AMs that it triggers using the circumplex model of emotion [32]; where the emotional content of both music and memories is operationalised primarily by arousal and valence. [8–12,18,33–35]. Some studies have assessed more complex emotional responses related to remembering MEAMs [1], or to hearing memory-evoking songs [36]. However, the current study was the first to characterise emotional experiences reported in memories as rich categorical emotion states, and to examine how those emotional experiences in these MEAMs may be influenced by features of the music in question. With suggestions that there exist between 20 and 30 distinct emotional states [37], and that emotion categories drive reports of emotional experience more than dimensions of arousal and valence [14], the extent to which such memory types can be predicted by musical stimuli constituted an important outstanding question.

Our results, which showed auditory profiles of memory-evoking music to be predictive of several complex categorical emotions – aesthetic appreciation, calmness, adoration, amusement, excitement, sadness and romance – demonstrate the relevance of this approach. We were able to show the distinct kinds of positive emotional experiences that music tends to be associated with, expanding on the established evidence that MEAMs are regarded as positive [38]. That joy, adoration, admiration, aesthetic experience and excitement were among the top most commonly reported emotions associated with AMs highlights that MEAMs may overlap with what is referred to in music psychology as strong experiences with music (SEM), understood as instances of music-listening involving high absorption, emotional intensity and ‘peak experience’ [39]. Most importantly, however, by demonstrating that musical features can predict the incidence of AMs with rich and mixed emotional qualities (as well as the phenomenological characteristics of these AMs), we show the potential for successfully evoking memories of specific kinds, for example in the context of reminiscence therapies for populations with memory impairments.

In any case, we show how the nuance of the emotionality of MEAMs may be lost upon reducing emotion into two dimensions of arousal and valence. Our findings show that while MEAMs are indeed generally positive, (as we found MEAMs to be described as positive on average (M = 3.86 out of 5), the nature of positive emotions associated with the MEAM may differ qualitatively with respect to the musical profile of the memory-evoking song. If not for the inclusion of complex categorical emotions, such distinctions would have been harder to detect. While some categorical emotions can be mapped to arousal and valence in predictable ways (i.e., excitement as high arousal-high valence and calmness as low arousal-high valence), others such as romance are less obvious. It follows that work concerning music-evoked autobiographical memory benefits from exploring the kinds of specific emotion states that are prevalent in AMs and the features of music that are able to cue such memories.

### Music features can predict phenomenological characteristics of AMs

Our study asked whether phenomenological characteristics of AMs such as vividness of remembering, importance to the individual, degree of social content of the memory, and uniqueness can be predicted by the musical cue, and we showed this to be case.

We demonstrated that memories of experiences that are highly social tended to be associated with less acoustic and more energetic, danceable (low E-A) songs, whereas memories of vivid, unique and self-important experiences were associated with more acoustic, contemplative (high E-A) songs. These results are in line with previous studies which have reported that positive music stimuli (danceable being a common characteristic) cue more social and energising memories compared to more negative (less upbeat) music, and that less arousing music elicits more vivid and unique memories than high arousing music [10].

Interestingly, a closer look at the phenomenological characteristics of MEAMs corroborate assumptions about the types of events or experiences at which certain types of music might be present and which may thus contribute to autobiographical encoding. In other words, our study shows that the nature of the match between music and AMs is consistent with social and cultural expectations. Parties, social events and nightclubs are likely to have energetic music playing, as well as dancing and socialisation. More melancholic, slow songs are unlikely to be present at these kinds of events, and consequently more likely to be associated with instances of independent or small group listening. Here, our finding that low energetic-high acoustic music was associated with occurrence of words relating to hearing are in line with this interpretation, as it indicates that more acoustic and sadder songs are associated with AMs characterised by attentive music-listening.

Our results additionally highlight that participants may evaluate MEAMs as positive even when they describe the initial event itself as being negative. We found that while low energetic-high acoustic songs were more likely to be associated with memories that had more negative words in their descriptions, this did not equate to a more negative AM valence rating. In other words, while AMs evoked by acoustic songs may be around sadder events, even these MEAMs overall are still considered by participants to be positive. This seemingly conflicting finding of ours is interesting as it speaks to the power of music to help listeners cope with negative events and appraise them in a more positive light. It is also consistent with a large body of work that emphasises MEAMS as being generally positive [8,25,33]. Once again, it seems important to note that this finding would not have been possible had we not accessed both their description of the event around the memory (allowing us to extract actual memory valence using LIWC) and their subjective evaluation of how the memory felt at the time (necessarily influenced by appraisal processes).

### The limits of music features’ predictive power

Our main analyses examining how features predicted qualities of memory mostly collapsed across self-selected and experimenter-cued memories. However these are objectively different types of memories due to several defining differences. In the former case, there is greater diversity in terms of genres, time periods and musical styles of the memory-evoking music, as well as an expectation that either those memories or songs reported might be more familiar or rehearsed compared to experimenter-cued MEAMs. Additionally, in self-selected MEAMs, musical stimuli are not acoustically present while for experimenter-cued MEAMs, they are.

Because of these differences, and even though the presence of music may not necessarily lead to more emotional recollections compared to other memory-evoking cues [40], a conservative approach to looking at how well music features of songs predict qualities of memories was taken whereby we only examined those memories where music was not heard during retrieval. Carrying out these analyses confirmed that some emotional qualities (arousal, calmness, excitement, negative words) and the phenomenological quality of social content continued to be predictable even with the substantially smaller sample of self-selected (N = 214) (compared with the combined sample (N = 1438) of MEAMs initially used), suggesting that our conclusions on the combined sample are largely supported. However, smaller sample size notwithstanding, it is relevant to consider which effects failed to be maintained when only looking at self-selected MEAMs: these less reliable effects were categorical emotions of aesthetic appreciation, adoration, amusement, sadness and romance; phenomenological characteristics like vividness, importance and uniqueness, high word count, age at time of memory, and finally, likelihood of using ‘hear’-related words in descriptions.

We suggest that these observed differences in E-A’s predictive ability of certain memory qualities may at least in part be due to characteristics unique to self-selected MEAMs. Self-selected MEAMs on the whole were associated with higher ratings for emotional and phenomenological variables, were likelier to be recollections of specific events compared to experimenter-cued MEAMs, and were also characterised by higher E-A values, (i.e., more acoustic and less energetic songs compared to experimenter-cued MEAMs). Indeed, all of these might have reduced the capacity for E-A to predict memory characteristics due to reduced variance and ceiling effects in both the predictor (E-A) and the variables (emotionality and phenomenological variables) we were examining.

Another possible explanation of the observed differences in E-A’s predictive ability is that the predictive capacity of auditory features holds only when music is acoustically present. That is, hearing the music influences responding, and in the case of high E-A songs, evokes more complex emotions (e.g., aesthetic appreciation, adoration) that in turn prime memories of a similar nature. Here it is important to note that, if that is the case, one proposed implication of the current work, (namely being able to predict memories evoked based on music features alone) may be more limited in scope than thought. Further studies will be needed to examine this more carefully.

In any case, at this point of considering different possible retrieval mechanisms in MEAMs, it is interesting to note that the degree of personal relevance music has for a listener (liking and familiarity) seems to be more powerful than acoustic features in predicting memories’ vividness, importance and uniqueness as well as some more complex emotions of aesthetic appreciation, adoration and romance. This finding corroborates previous findings showing a strong influence of liking and familiarity on memory qualities and also reinforces the idea that, when available, such information (about preferred or personally salient music) is especially useful in ensuring that vivid and important memories are elicited [41].

### Implications, further directions and limitations

#### Implications.

A compelling finding from the current research is that the tendency of certain music to be present in certain contexts may lead to it being a good trigger of particular types of memories. In other words, we show that music with certain features may be better able to conjure up specific moments in our past. At the same time, our results hint that more qualities of personally significant MEAMs (here self-selected MEAMs) may be less strongly predicted by the music’s acoustic-musical profile. Our study’s findings speak to the relevance of trying to bridge the divide between studying the role of music at encoding and studying the role of music at retrieval since neither the former nor latter alone can fully explain the mechanisms underlying music-related autobiographical memories.

Our study also speaks to the importance of broadening the way in which music and memories are characterised away from the 2-dimensional circumplex model. We found that in the context of familiar self-selected and Billboard songs, musical characteristics may be distilled into one main component to describe the music that is listened to in the (Western) population. In a study by de Fleurian & Pearce using Spotify auditory features to explore musical profiles of chill-evoking songs, a principal components analysis revealed a component similar to our E-A, characterised by high negative loadings for acousticness and instrumentalness, and high positive loadings for arousal, valence, loudness, danceability and tempo [16]. This outcome demonstrates how ecological validity of studies may be lost by forcing musical stimuli into one of four arousal-valence quadrants. Taken together, ours and De Fleurian & Pearce’s musical feature analysis appear to be picking up on this collapsed dimension which demonstrates that, particularly in music that participants identify as listening to out of choice, arousal and valence tend to be correlated.

Further, our results add nuance to the existing body of work as they show that a particular set of low- and high-level auditory features tend to group together and consequently relate to characteristics of MEAMs in reliable ways. We also show that some individual features are especially predictive of certain MEAM characteristics (tempo predicting uniqueness and importance, for instance), while others seem to relate minimally with MEAMs (speechiness, instrumentalness, liveness, key and mode). These findings are an interesting first step in looking at low- and high-level stimulus properties of memory-evoking music, and can help in shaping hypotheses for future research.

Finally, our findings that self-selected MEAMs differed in many ways from experimenter cued (were more specific, vivid, positive, arousing, unique and important than experimenter-cued MEAMs) demonstrate the importance of careful consideration of methodology and music stimuli used in evoking AMs. It seems clear that self-selected music is associated with more meaningful and especially episodic AMs, as has also previously been shown in populations with Alzheimer’s Disease when comparing self-selected to researcher-selected music and silence [41]. In any case, these findings highlight the role of music’s personal significance in MEAMs, and accordingly in the building of an identity and sense of self.

#### Further directions.

The present work provides new insights into potential mechanisms behind music-evoked reminiscence and how perceptual experience may drive memory of certain details and not others. However, understanding the factors that influence the degree of accuracy of MEAMs could be considered to lie at the frontier of MEAM research and has many interesting implications. The current body of work shows MEAMs to be experienced as vivid; however, with the majority of methods relying on self-report of events that occurred up to decades earlier, there is a strong need to improve our understanding of whether music contributes to actual accuracy of memories recalled or alternatively simply enhances feelings of vividness that are independent of coherence with the initial experience of the event.

On this topic, recent work by Jakubowski and colleagues has begun to demonstrate that music, compared to non-musical sounds, may lead to similarly vivid but less accurately recalled episodic memories. It will be interesting to examine the extent to which this observation holds in the case of long-term autobiographical memories [42]. In any case, future studies would benefit from carefully considering conditions of MEAM encoding and retrieval, with or without acoustically presented music, and how those factors may be influencing results.

#### Limitations.

It is worth noting that our study, as is the case with many studies of MEAMs relying on Billboard music to cue memories, is limited by the nature of the music cues. That is, the conclusions drawn from this work rely on a set of music stimuli that (with the exception of self-selected MEAMs) are all Western, contemporary, and chart-topping songs. Whether or not the same grouping of auditory features would continue to predict the same emotional and phenomenological characteristics of MEAMs as found here (had the music stimuli represented non-Western musical cultures) will need to be investigated. It is well-evidenced that culture influences several processes that relate to autobiographical memory such as emotion knowledge, self-goals and perceptual style [43]. Thus, it follows that a lack of both non-Western stimuli and participants limits a comprehensive and fully generalisable understanding of music-related AM processes and that future work will benefit from specific attention to MEAMs cued by a variety of genres and musical traditions.

## Conclusions

Our study showed that music features of memory-evoking music predict several characteristics of AMs. As such it extends established findings that expressed emotionality of music influences the kinds of MEAMs that are retrieved and the efficiency of their retrieval. It also extends findings that auditory features of memory-evoking music may impact AM through said emotionality. Our study adds nuance to existing research in key ways. Not only does it elaborate on a wider range of possible effects that objective stimulus features of music cues can have on evoked memories, but it also highlights some of the distinct kinds of emotional AMs that music may be able to evoke. Finally, it highlights the importance of introducing novel methods to study MEAMs: ones that reflect naturalistic experiences of MEAMs and ones that can begin to tease apart how different retrieval conditions may influence the nature of memories recalled.

## Supporting information

S1 Appendix Data Cleaning and Assumptions. In this supplementary appendix we report our complete data cleaning procedures and assumption testing for statistical models.
(DOCX)
