# Supplementary material for: Qualities of music-evoked autobiographical memories are associated with auditory features of the memory-evoking music
Source: PLoS One. 2025 Aug 20;20(8):e0329072. doi: 10.1371/journal.pone.0329072 (PMC12367148; doi:10.1371/journal.pone.0329072)

**S1 Appendix: Data Cleaning and Assumption Tests**

# Data Cleaning

## Dealing with missing music features

To acquire the musical features of reported memory-evoking music, two playlists were created on Spotify that listed all of the songs to be included in analysis, and musical feature data was acquired via the Spotify API which accesses the data of songs within each playlist. All Billboard songs that were included were found on Spotify, and so musical feature data were available for all experimenter cued songs. For self-selected songs, musical features were not able to be acquired for all songs, due to some participants either not providing enough details to identify the correct song (missing song or artist name), some participants listing entire albums/EPs, or Spotify not having the song available. In total, musical features of 19 self-selected songs could not be identified, so 214 of the originally 233 self-selected MEAMs were included in analysis.

## Dealing with NA values

Upon inspection one participant was identified who had empty responses; as such, this participant and their 11 corresponding MEAMs were removed from the dataset (note that this participant’s self-selected MEAM is one of the above 19 self-selected MEAMs that was not included).

An additional 16 MEAMs were identified in which participants indicated a memory but did not provide a description of memory or answer Likert variables questions (ratings for vividness, arousal, valence, uniqueness, importance and social content of memory). These entries were also removed prior to analysis. Further, across outcome variables, we identified NA values where participants skipped or did not provide responses for AM vividness (N = 10), arousal (N = 12), valence (N = 18), uniqueness (N = 8), importance (N = 13), social content (N = 10), age at time of memory (N = 8), and spontaneity (N = 18). NA values for each variable were imputed to the mean of that variable.

These steps resulted in the removal of 45 entries, for a dataset of 1438 MEAMs that would be included in subsequent mixed effects models.

## Dealing with outliers

For the continuous outcome variables (excluding retrieval time as range for retrieval time was limited between 0 and 20 seconds) we identified univariate outliers as entries with z-scores > |3.29|, and removed those entries prior to analysis. Below shows the number of outliers removed for each variable that was identified as having outliers. Subsequent analyses for these variables were conducted on datasets excluding those outliers.

| **Variable** | **Number of outliers removed** |
| --- | --- |
| memory age | 21 |
| hear | 11 |
| motion | 18 |
| negemo | 10 |
| posemo | 23 |
| see | 32 |
| social | 15 |
| space | 13 |
| feel | 32 |
| word count | 3 |

# Assumption Tests

## Linear Mixed Effects Models

Linear mixed effects models (LMEMs) are robust to violations of normality (Schielzeth et al., 2020); however, models are expected to meet the assumption that residuals are normally distributed. To check this assumption, Q-Q plots were generated for all LMEMs (see below). We initially planned to evaluate LIWC outcomes using LMEMs but inspection of Q-Q plots for these models revealed residuals to not be normally distributed, partially due to high prevalence of 0 value entries. Consequently, we treated these as logistic regressions by creating new binomial variables for each LIWC category, assigning either a 0 (for 0 value-entries) or a 1 (value is above 0). For word count, we used a median split to assign 0 and 1 values instead, as the issue with high 0 value entries did not apply for this variable.

Inspection of Q-Q plots revealed memory age and memory valence, social content, and spontaneity or recall may not meet the assumption of normality of residuals. We performed non-parametric Spearman correlations to assess non-linear relationships between PC1 and these identified outcome variables. For memory age, we averaged PC1 values across age to minimize over-reporting of results. The same averaging didn’t apply as the remainder were Likert questions. Results are below:

- Memory age : t = 14.57, df = 1434, p < 0.001
- Valence: t = -0.77, df = 1434, p = 0.44
- Social: t = -4.03, df = 1434, p < 0.001
- Spontaneity: t = -1.91, df = 1218, p = 0.06

These results of the non-parametric tests demonstrate the same relationships as observed for our LMEMs (significant positive effect of PC1 on age of memory, negative effect of PC1 on social, and no significant effects on valence and spontaneity). Based on these results, Q-Q plots not appearing to be too strong violations of normality of residuals, and the motivation to account for participant and song effects, we proceeded with LMEMs for these variables as planned.

## Mixed-effects logistic regressions

For logistic regressions, we inspected plots of log-odds of outcome variables against the predictor PC1 to assess if models met the assumption of linearity of the log-odds. Upon inspection we identified that models for amusement, excitement, interest, satisfaction, awkwardness may not meet the assumption. Other models for LIWC variables and other categorical emotion variables appeared to meet the assumption. For those that were identified as potentially not fitting the assumption, we ran Box-Tidwell tests to confirm whether assumption of linearity of logit was met. For excitement, interest, satisfaction awkwardness, and amusement models, the Box-Tidwell test confirmed that the assumption of linearity of the log-odds was met. There was an error in the fit for the model for amusement, with error message saying that there were NA, NaN, or Inf values, but inspection revealed this to not be true. In order to run the Box-Tidwell test, we ran the logistic regression without random effects which revealed very similar results to our linear mixed effect model (Estimate = -0.31, SE = 0.04, z = -7.14, p < 0.001), and used this model for the Box-Tidwell test, which confirmed the assumption of linearity of log-ods for this model.

# Q-Q Plots

*Memory age*


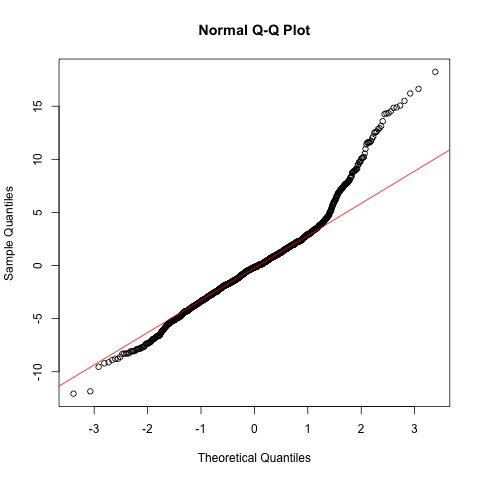


*Memory vividness*


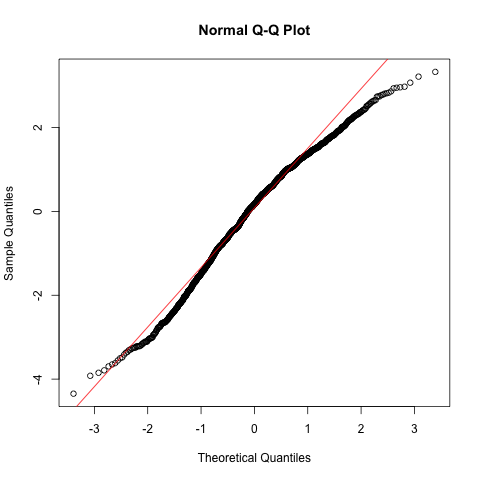


*Memory valence*


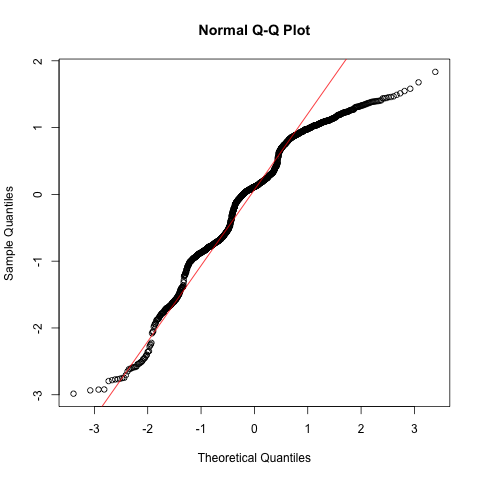


*Memory arousal*


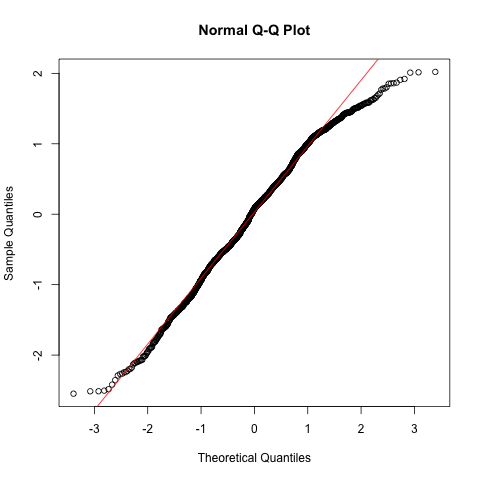


*Memory uniqueness*


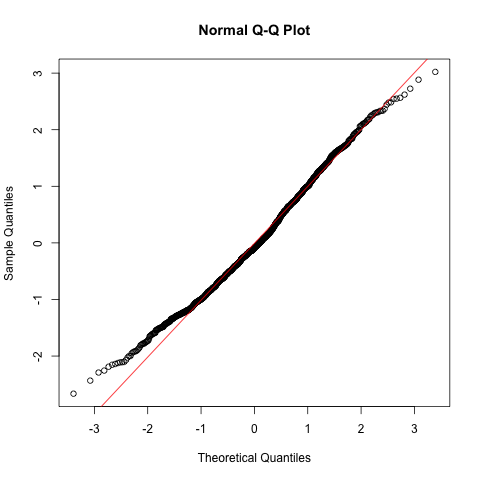


*Memory importance*


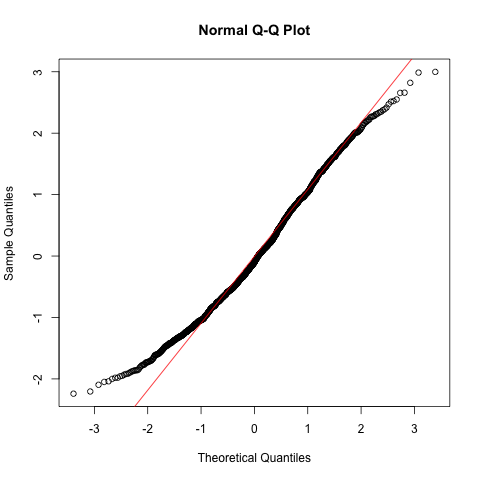


*Memory social content*


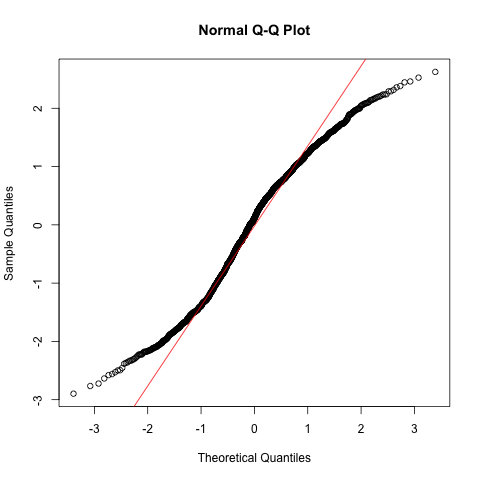


*Retrieval time*


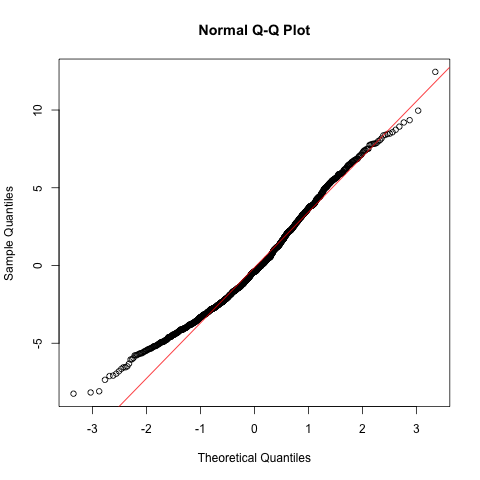


*Spontaneity of recall*


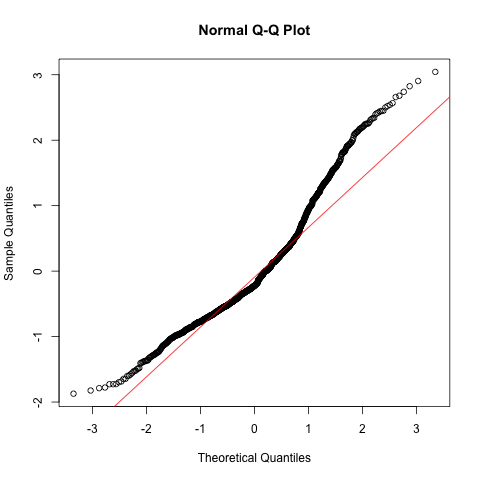


*LIWC Posemo score*


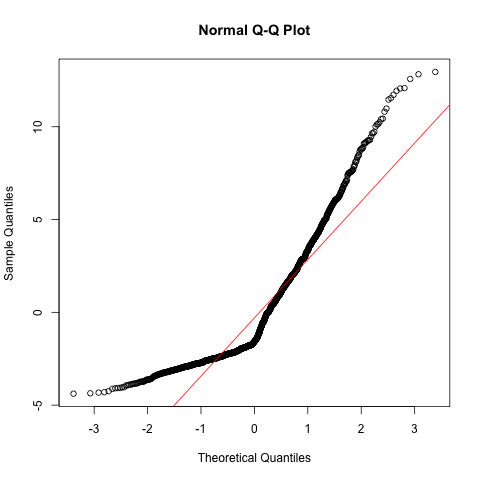


*LIWC Negemo score*


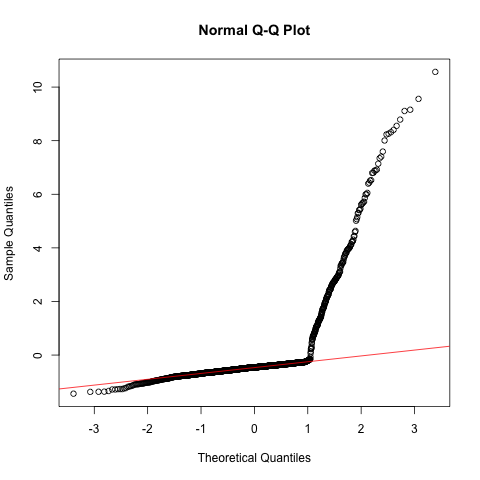


*LIWC Motion score*


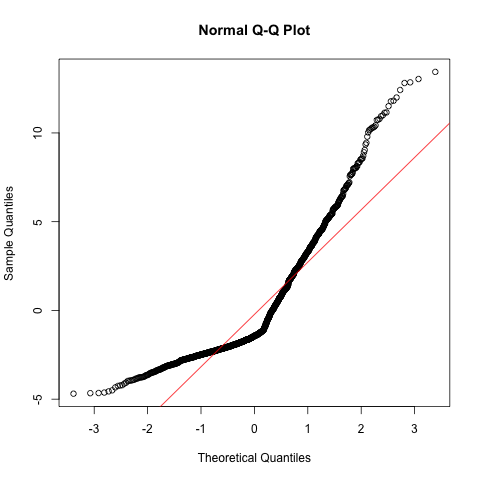


*LIWC space score*


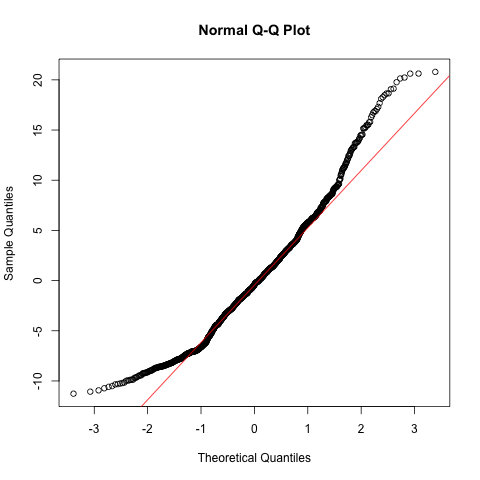


*LIWC hear score*


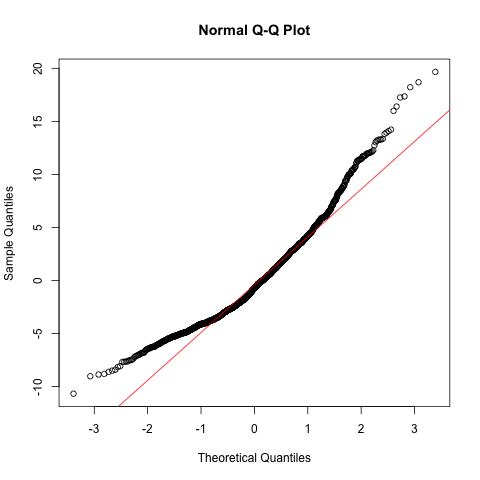


*LIWC social score*


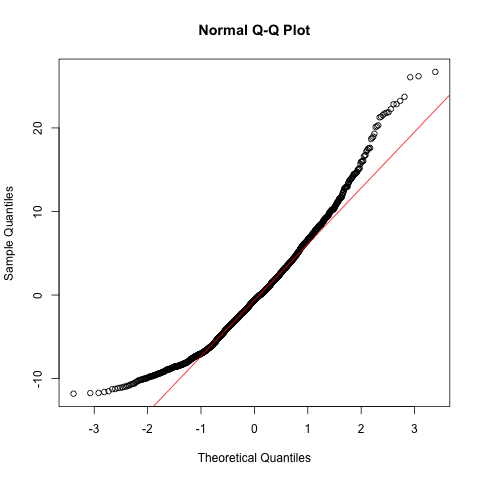


*LIWC word count*


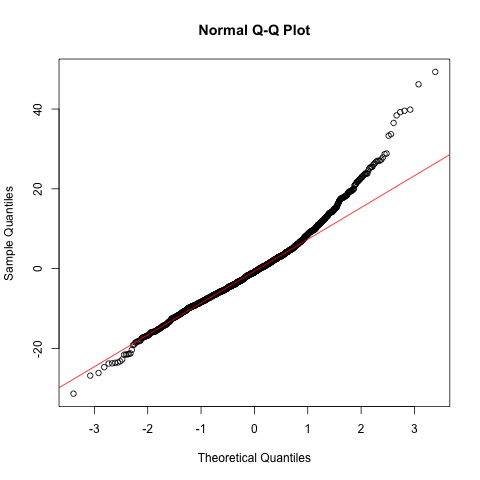


# Plots of log-odds against predictor

*Word Count*


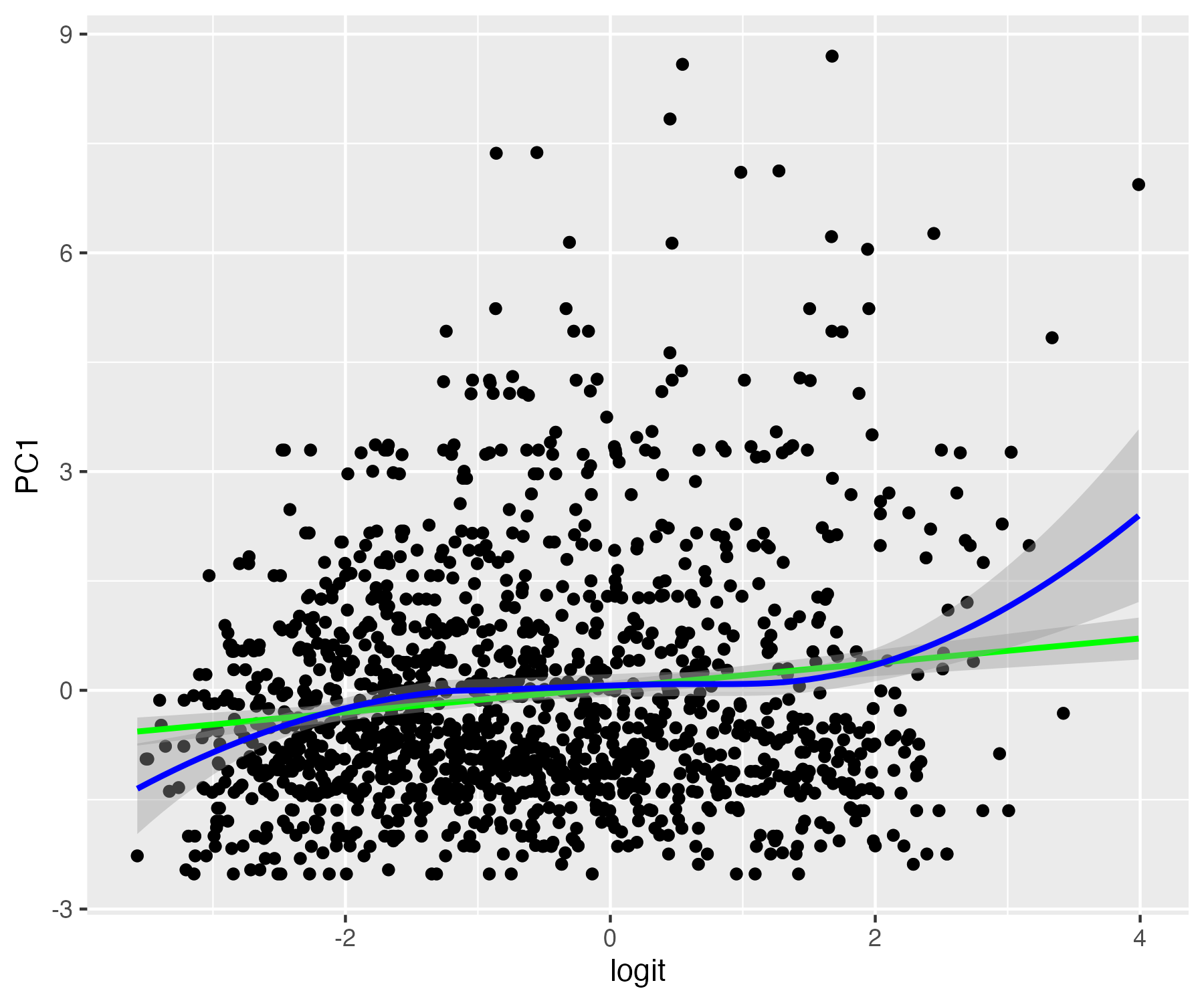


*LIWC Posemo*


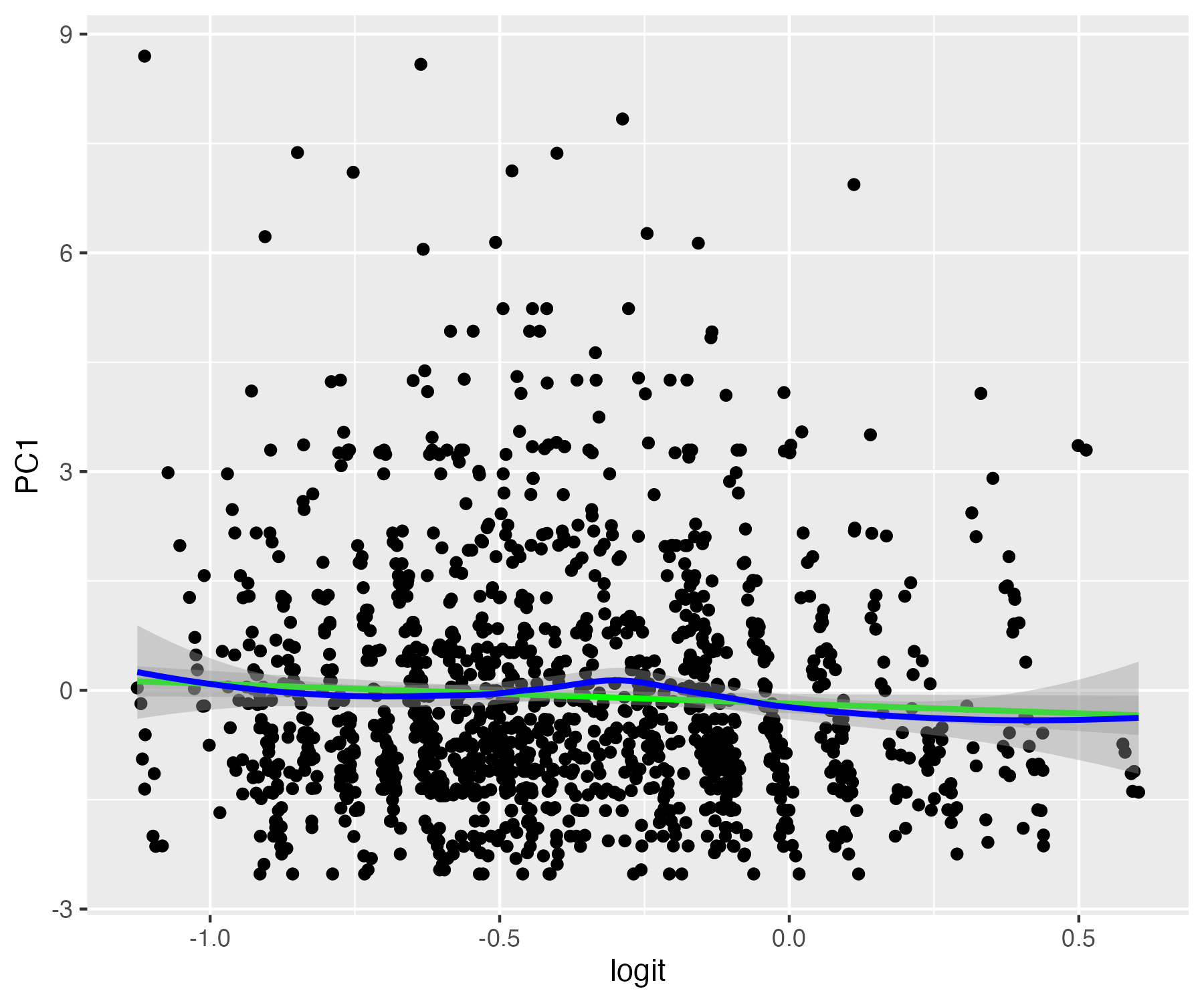


*LIWC negemo*


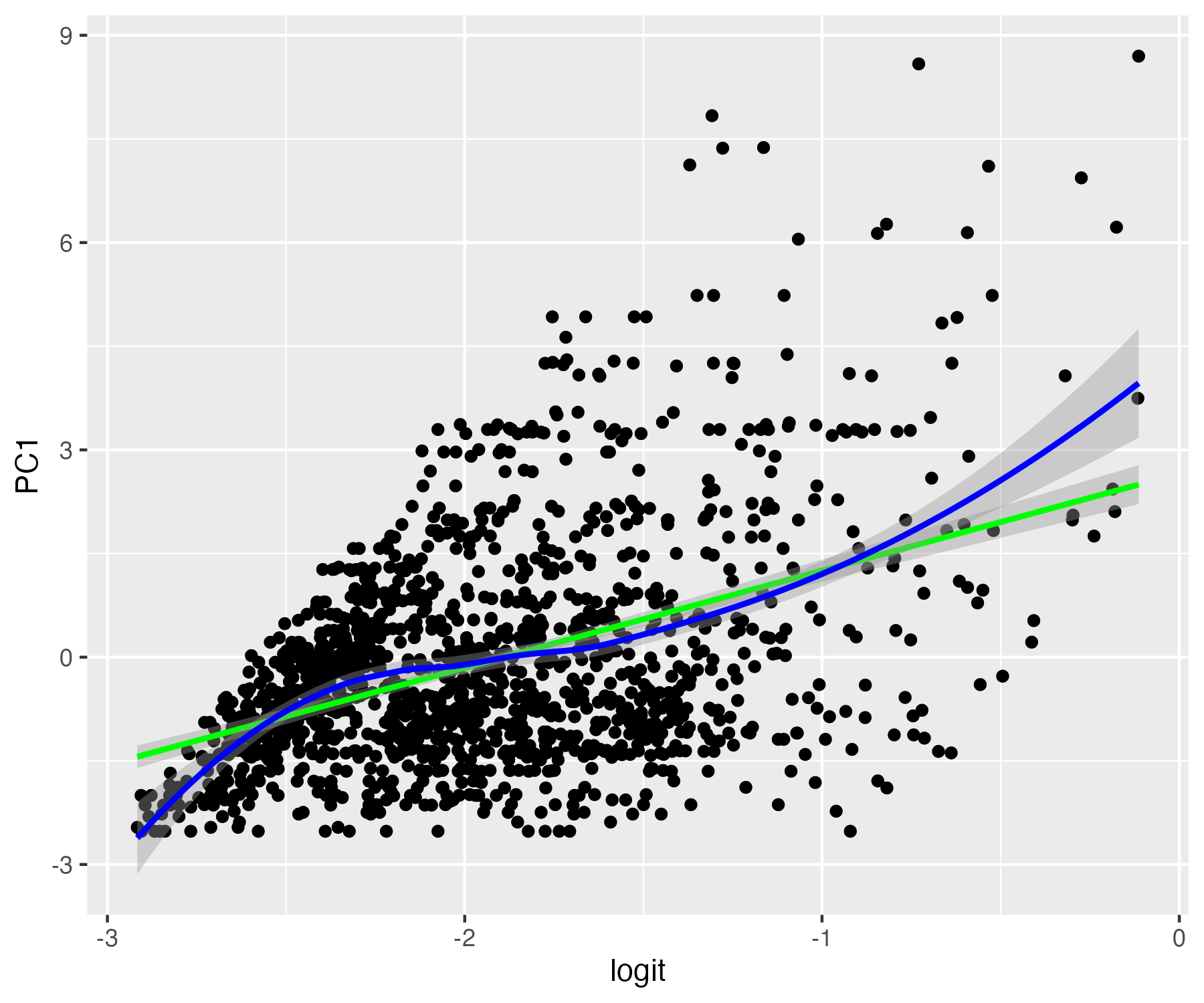


*LIWC motion*


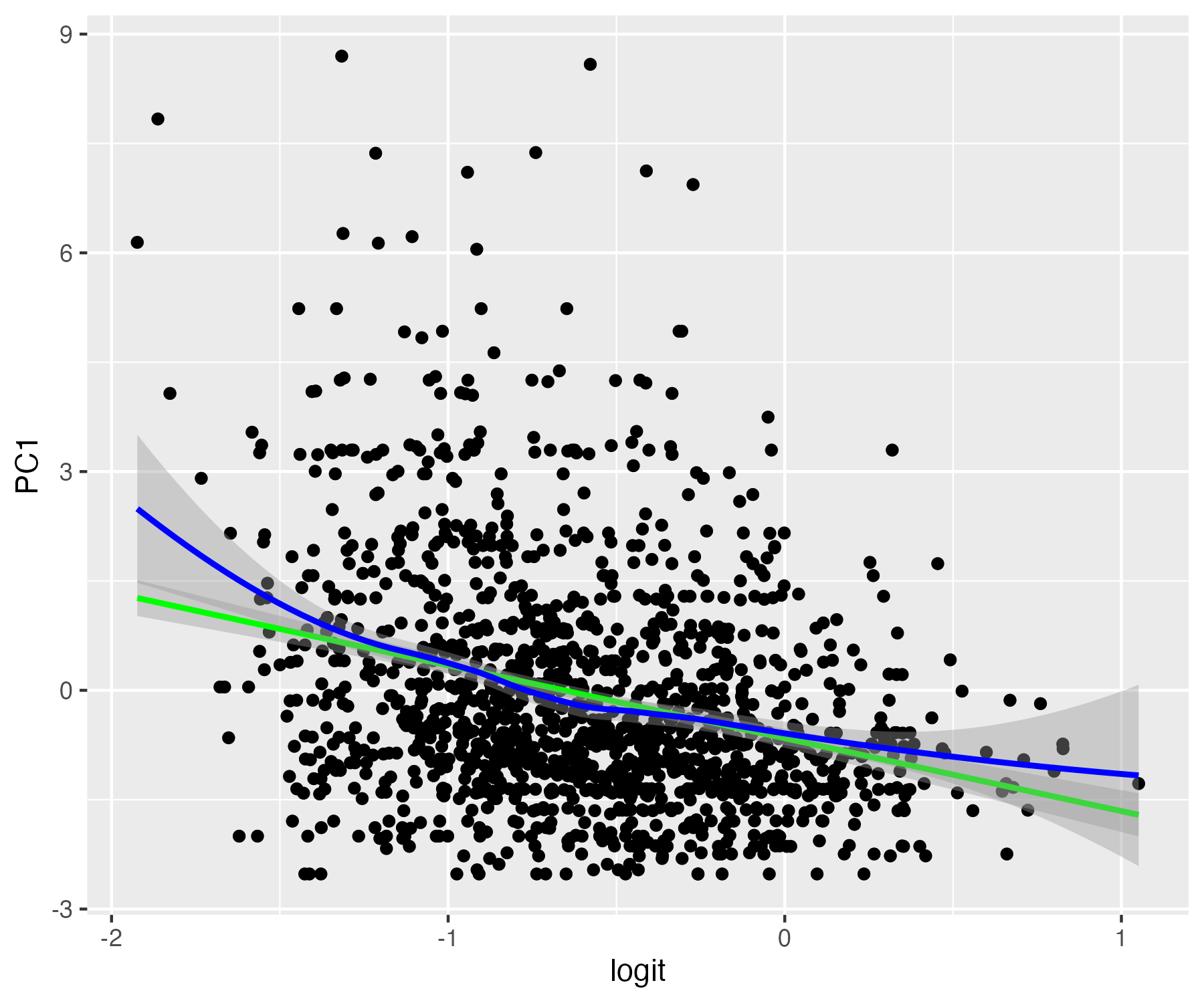


*LIWC space*


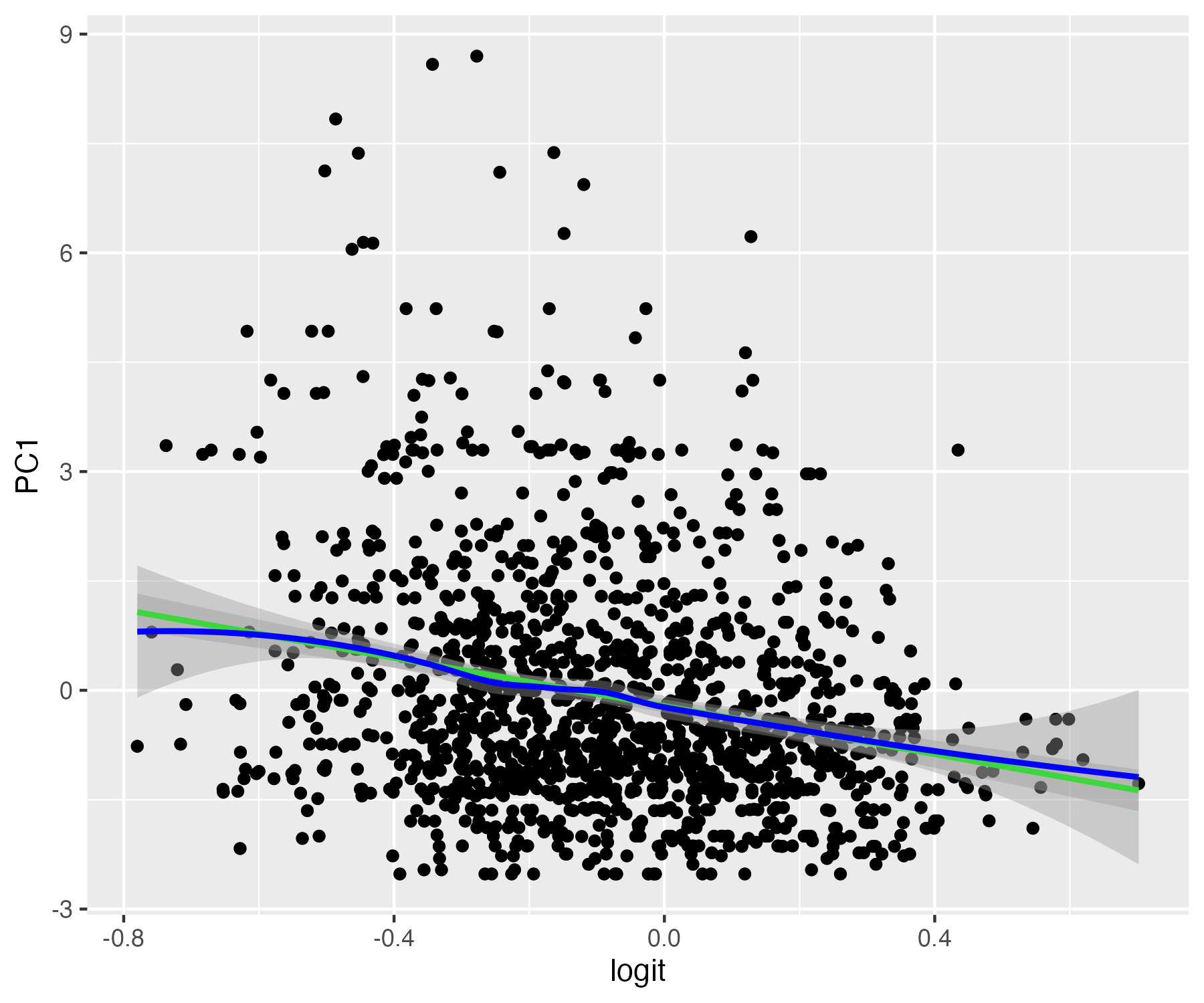


*LIWC see*


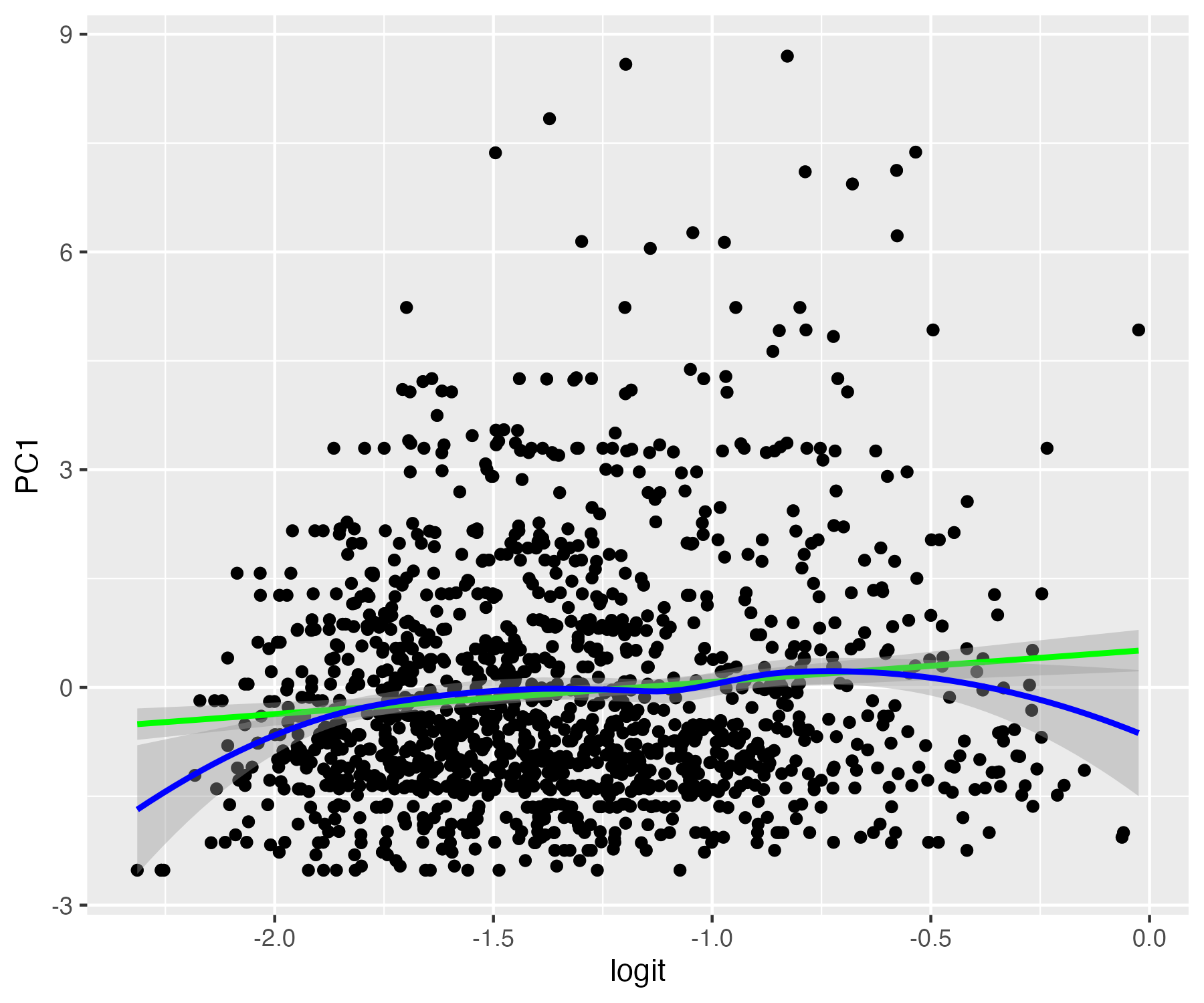


*LIWC hear*


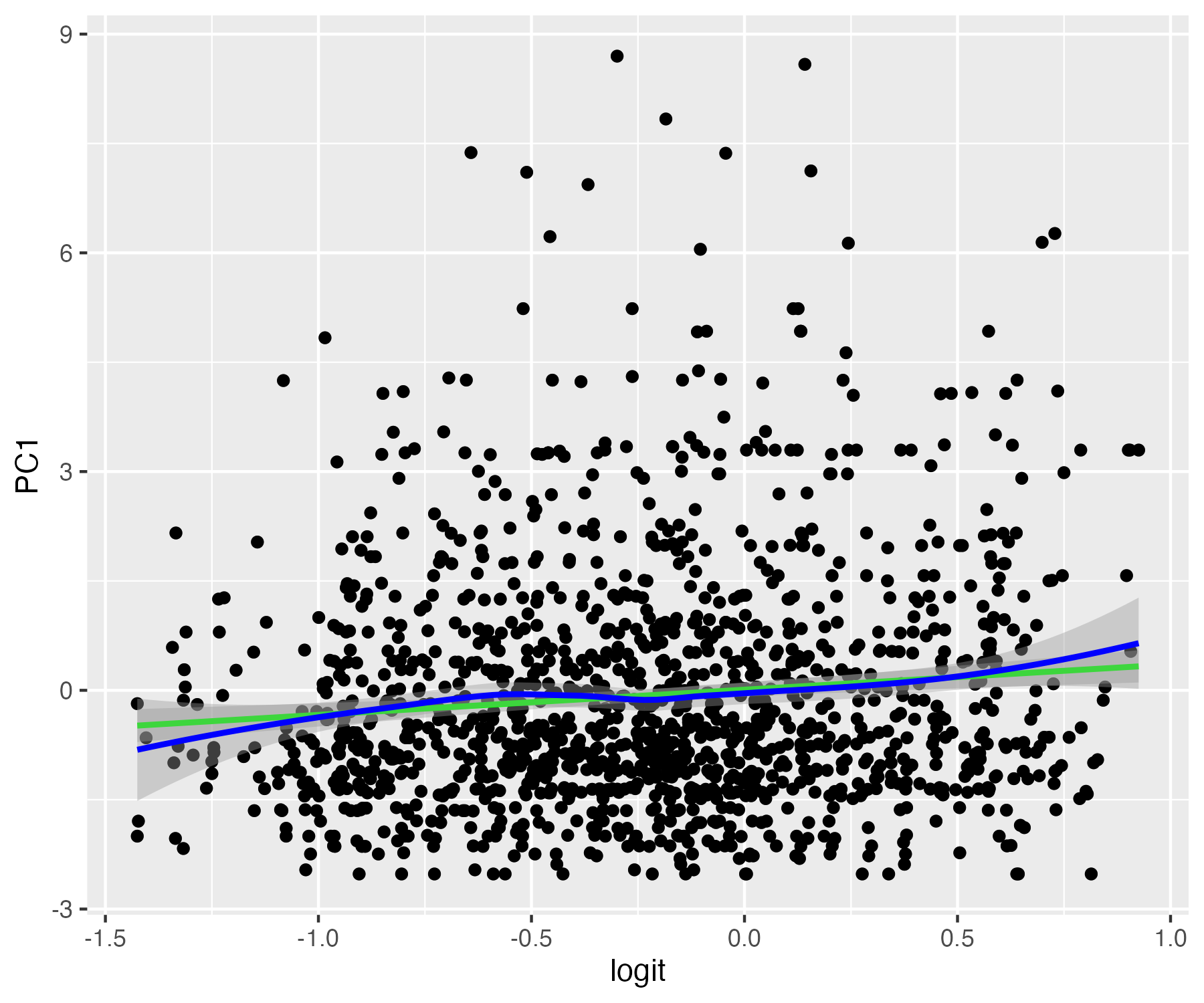


*LIWC social*


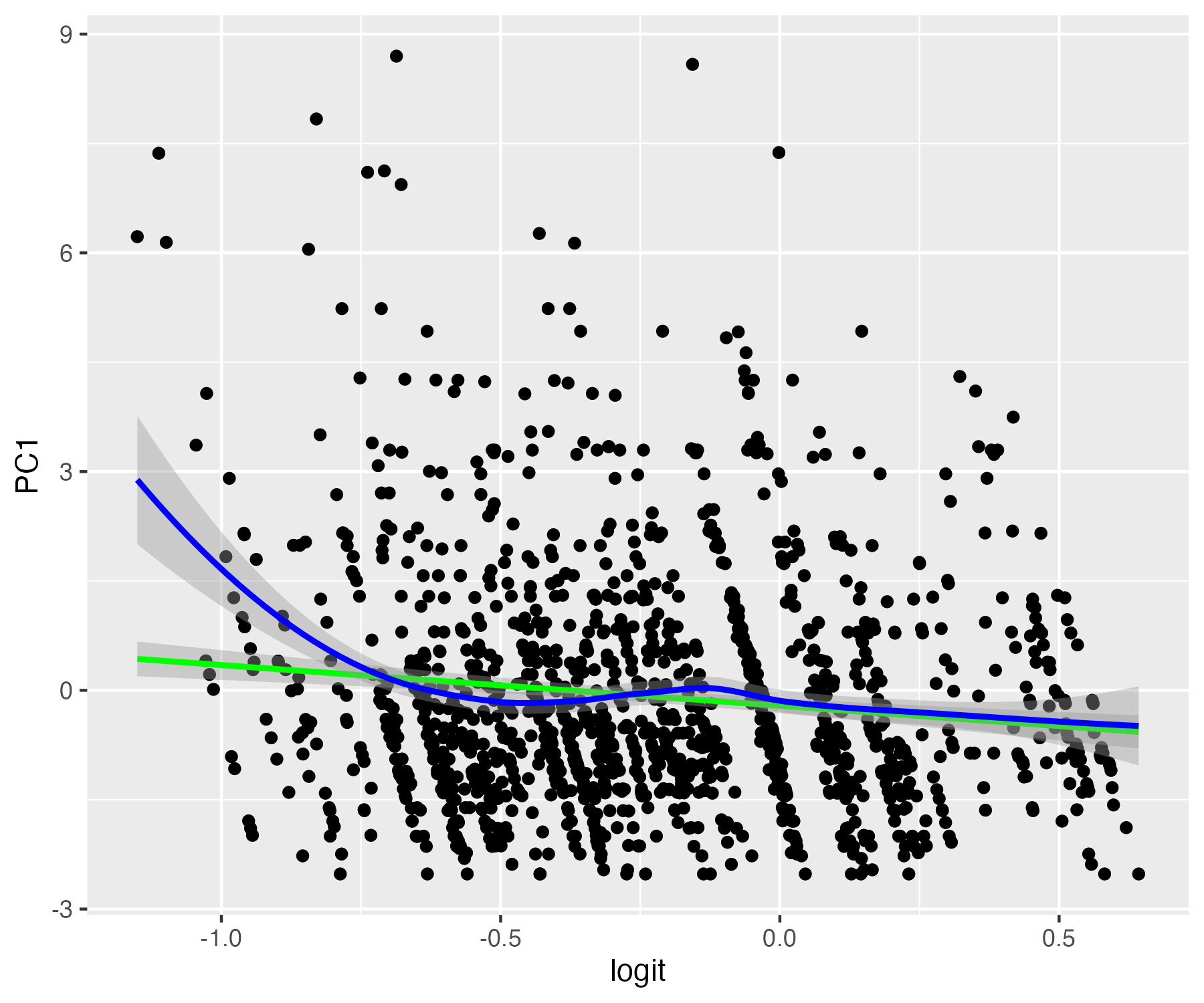


*LIWC feel*


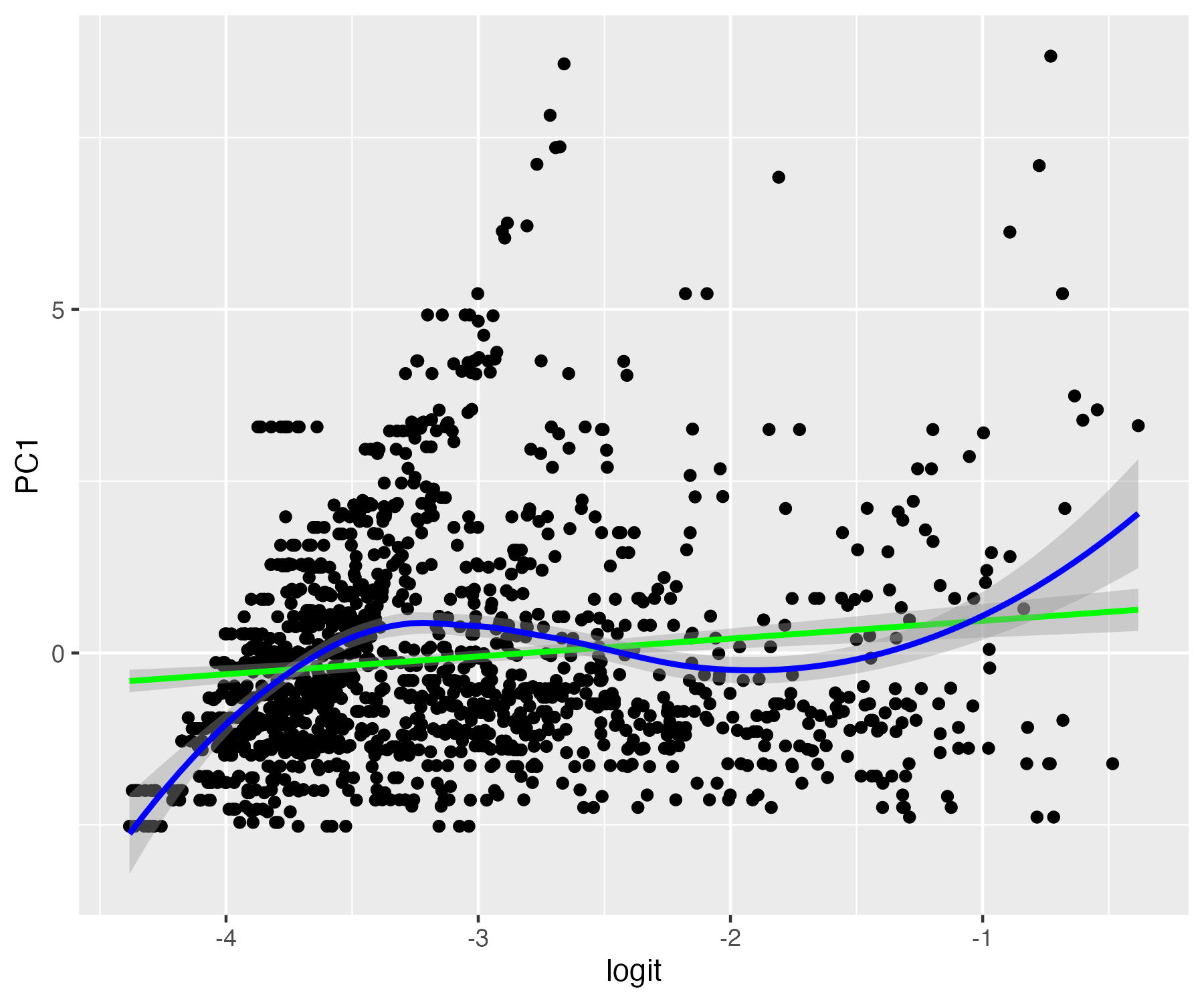


*Admiration*


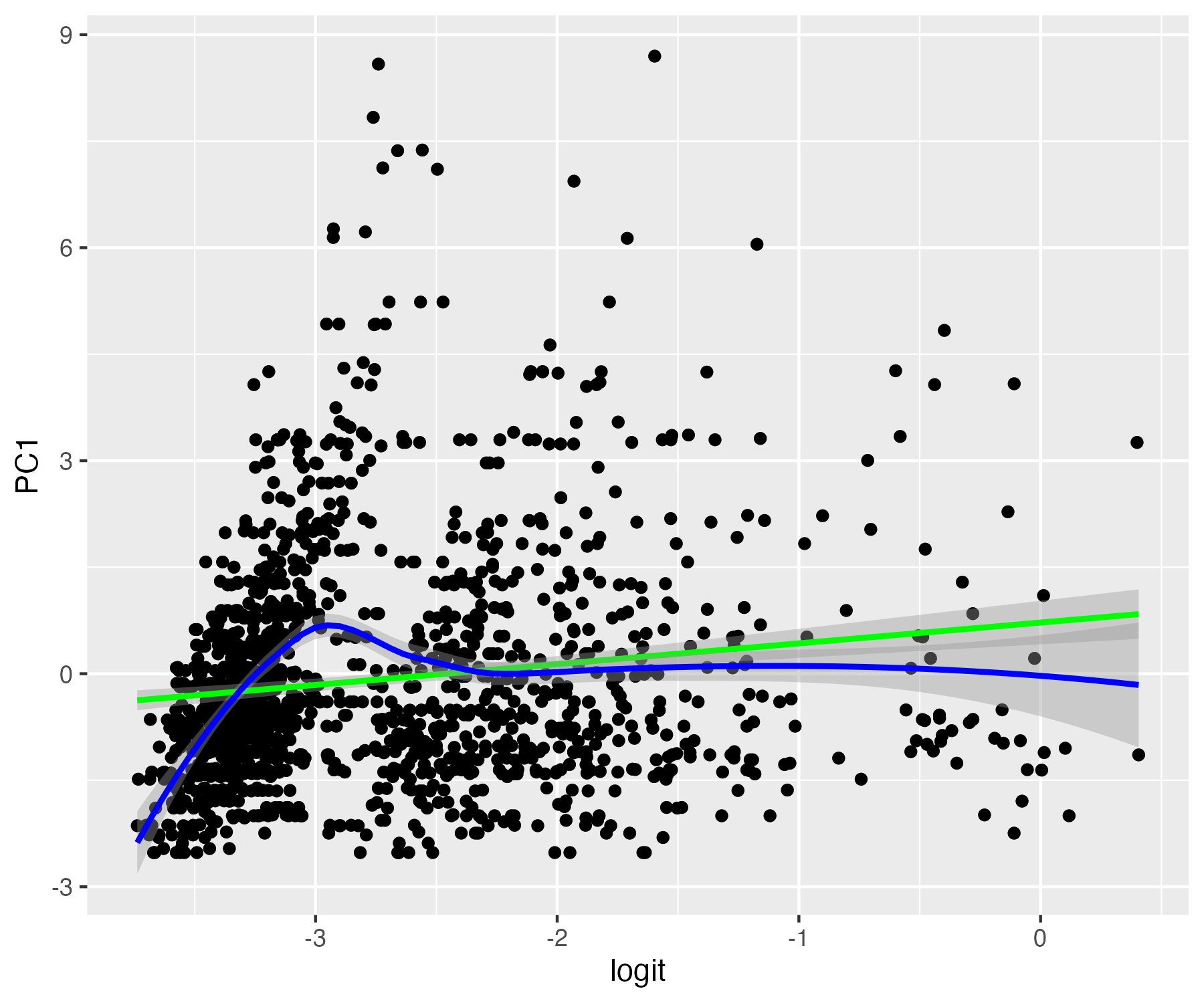


*Adoration*


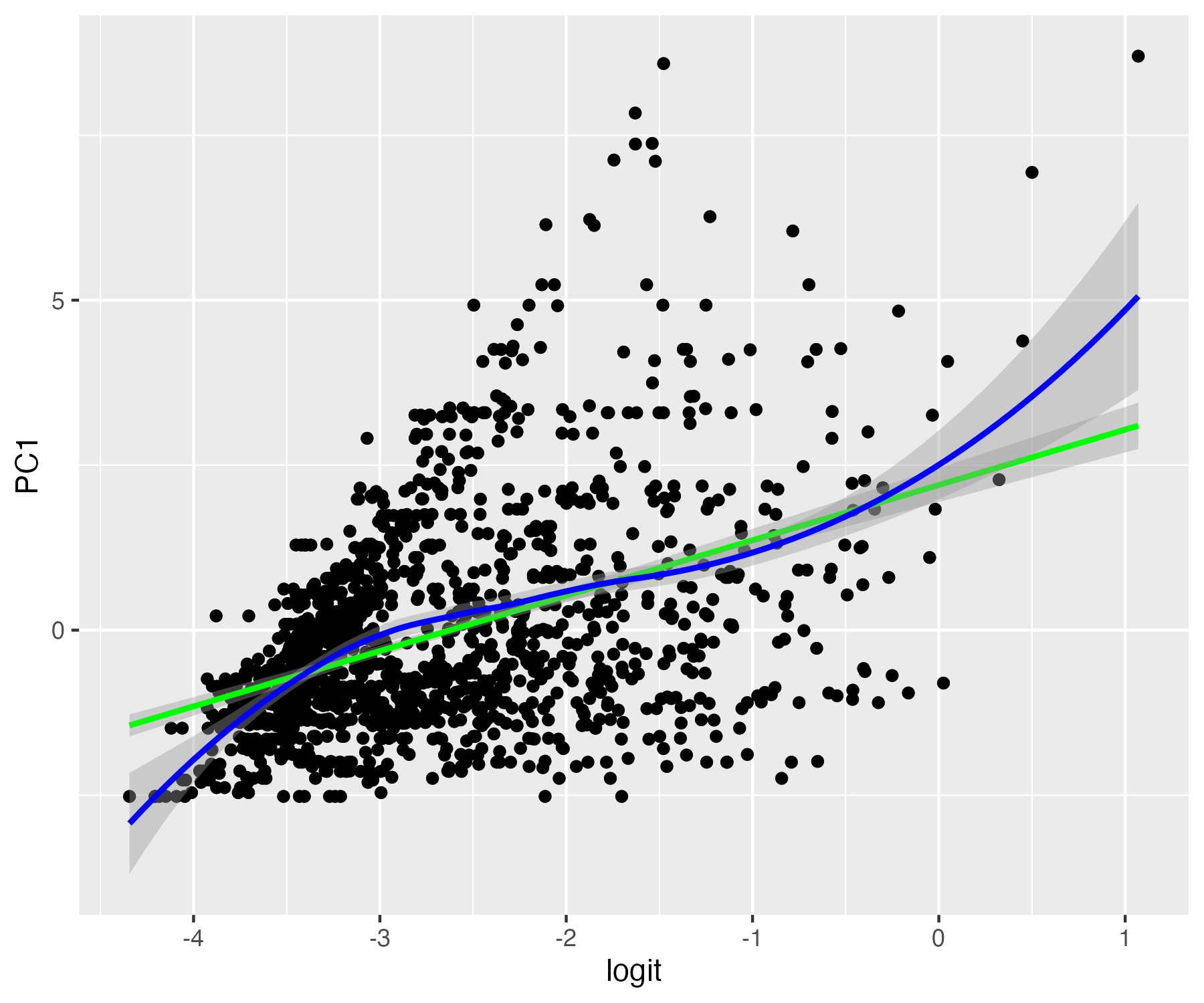


*Aesthetic appreciation*


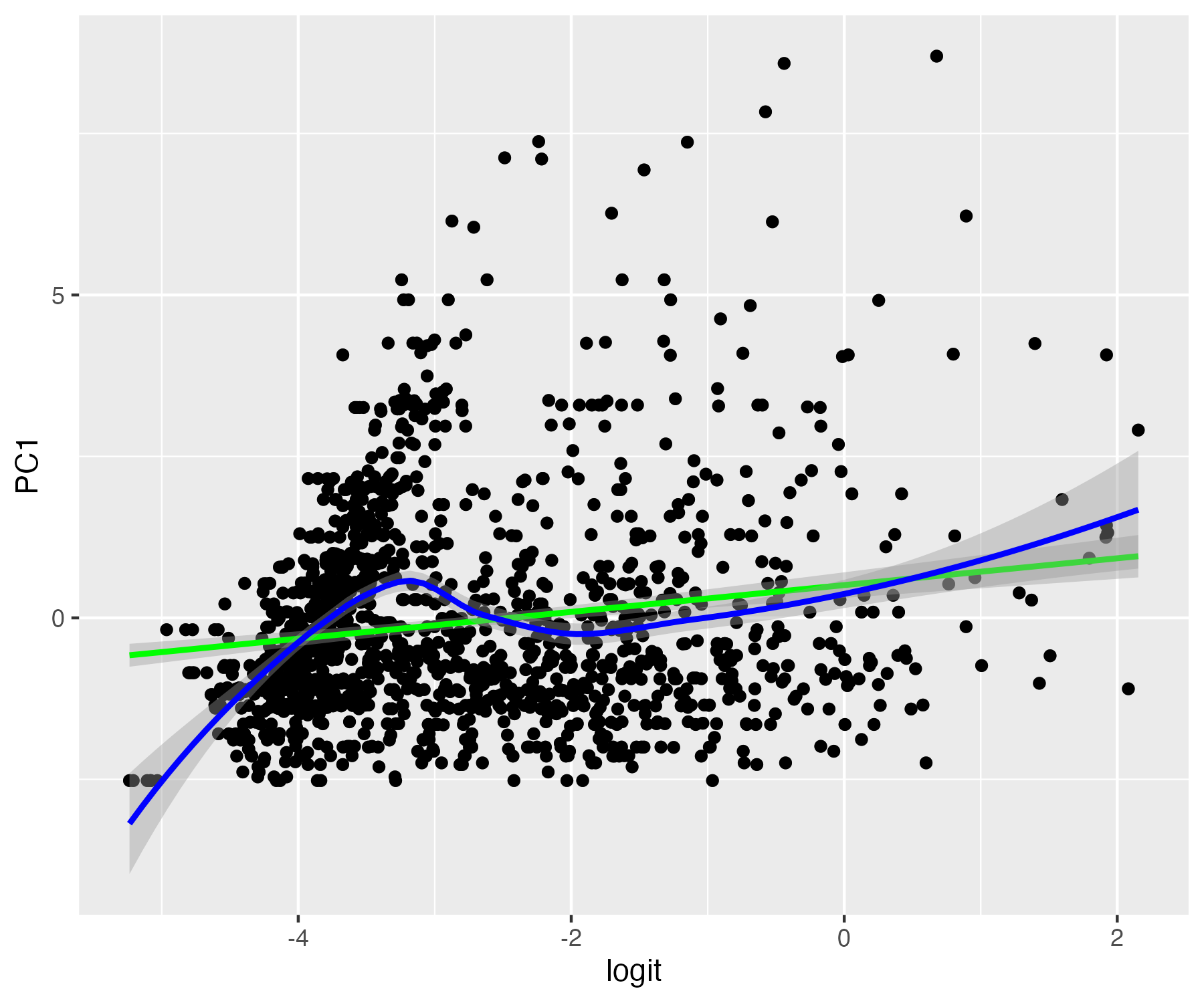


*Amusement*


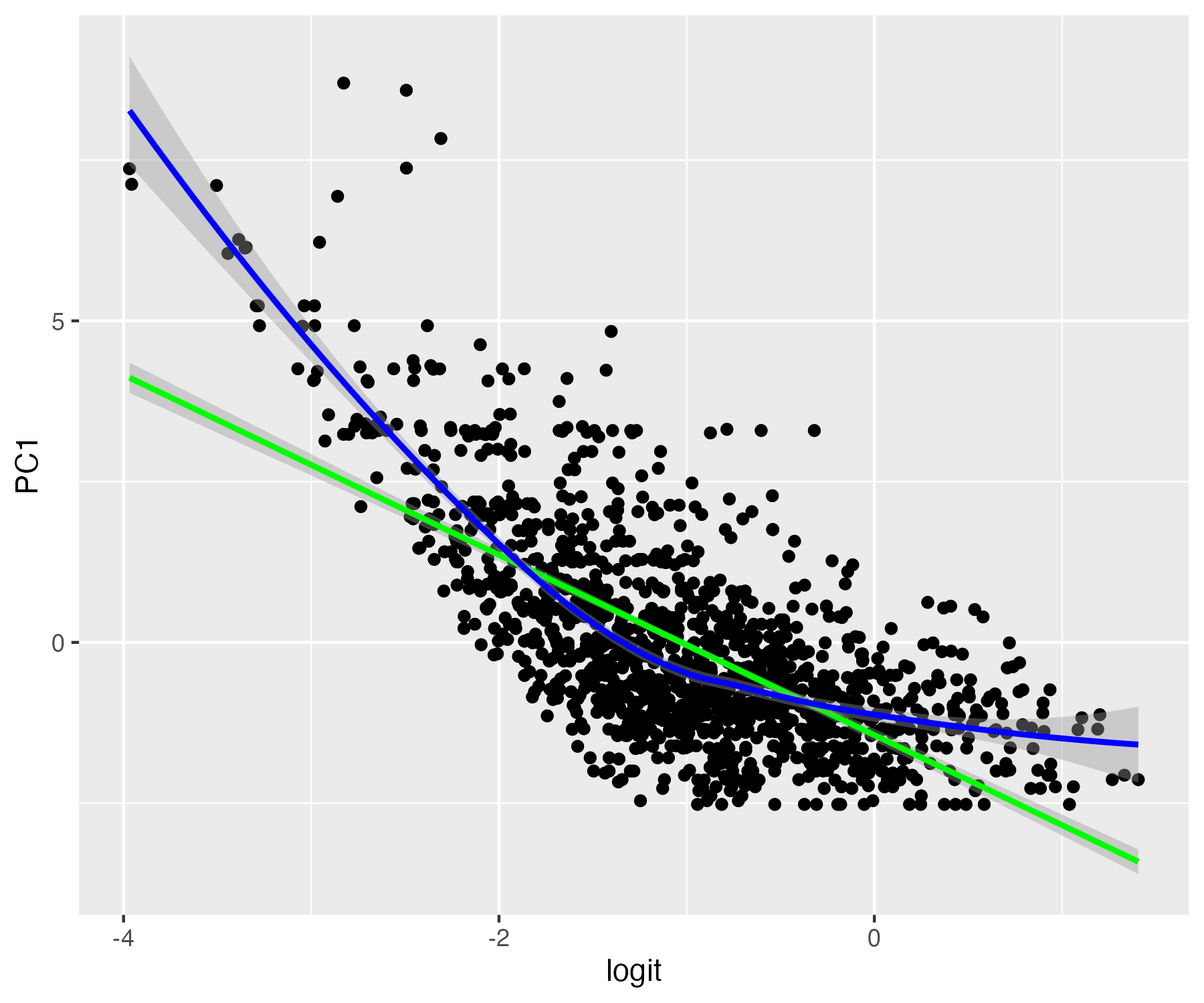


*Calmness*


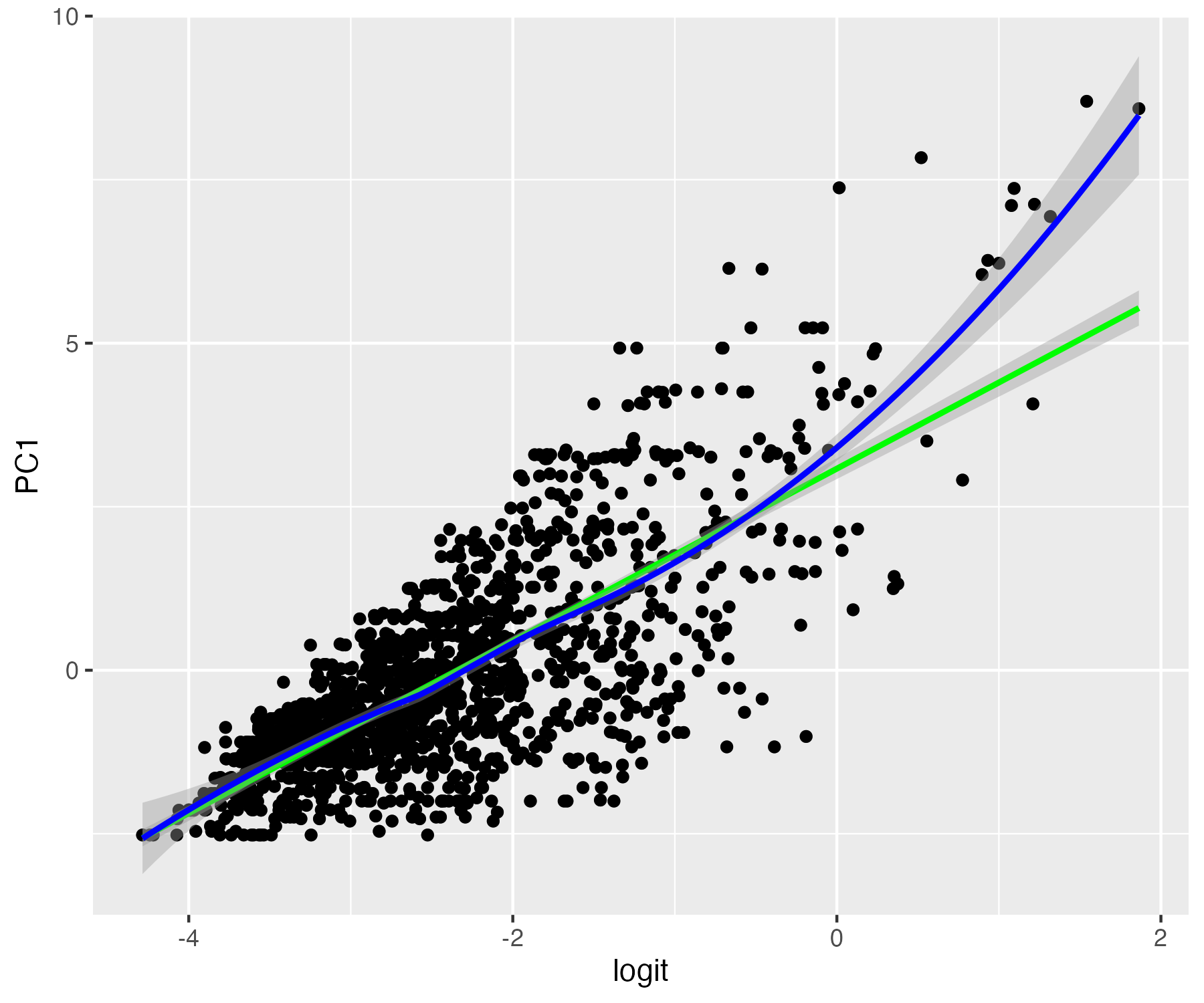


*Excitement*


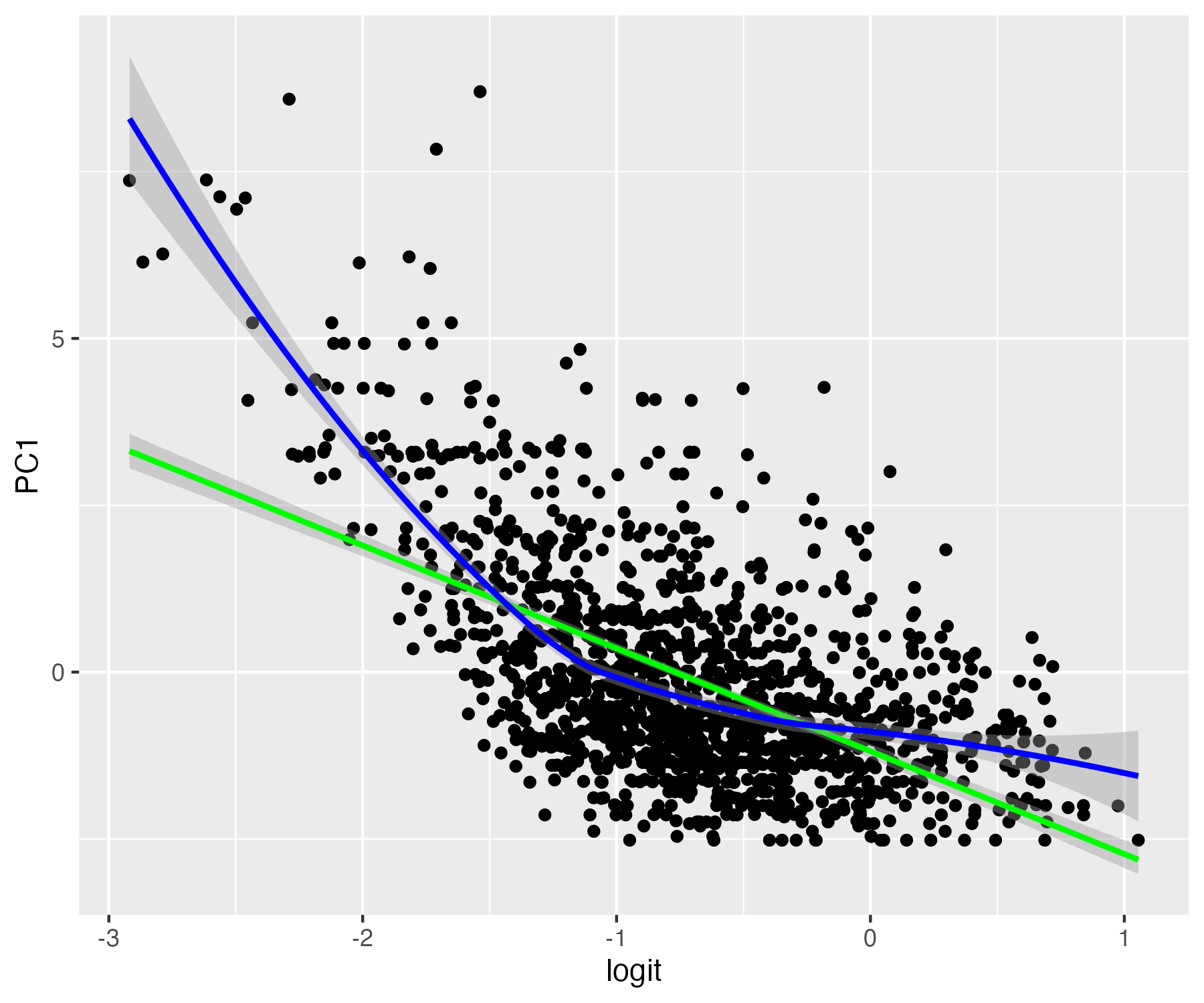


*Interest*


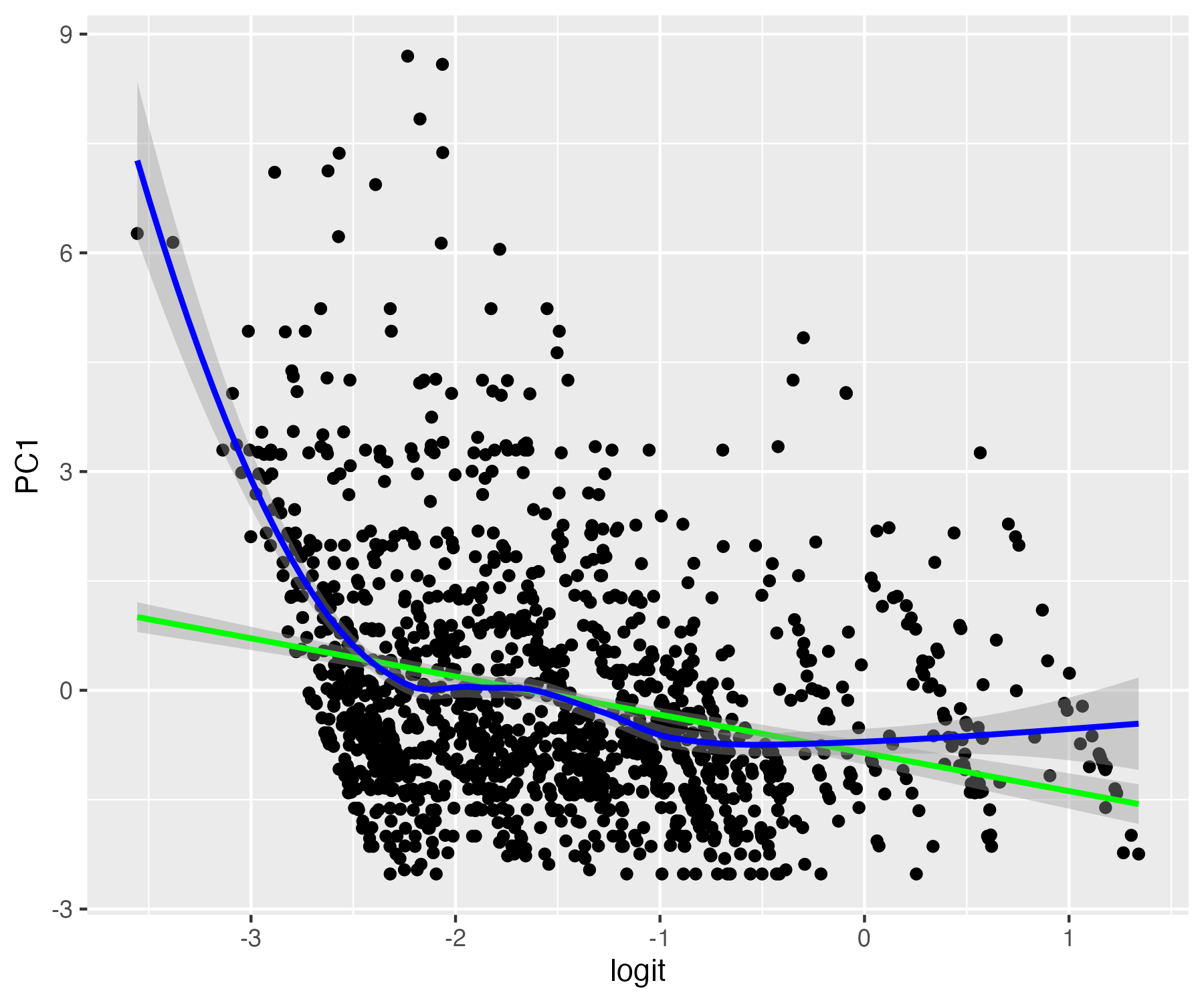


*Joy*


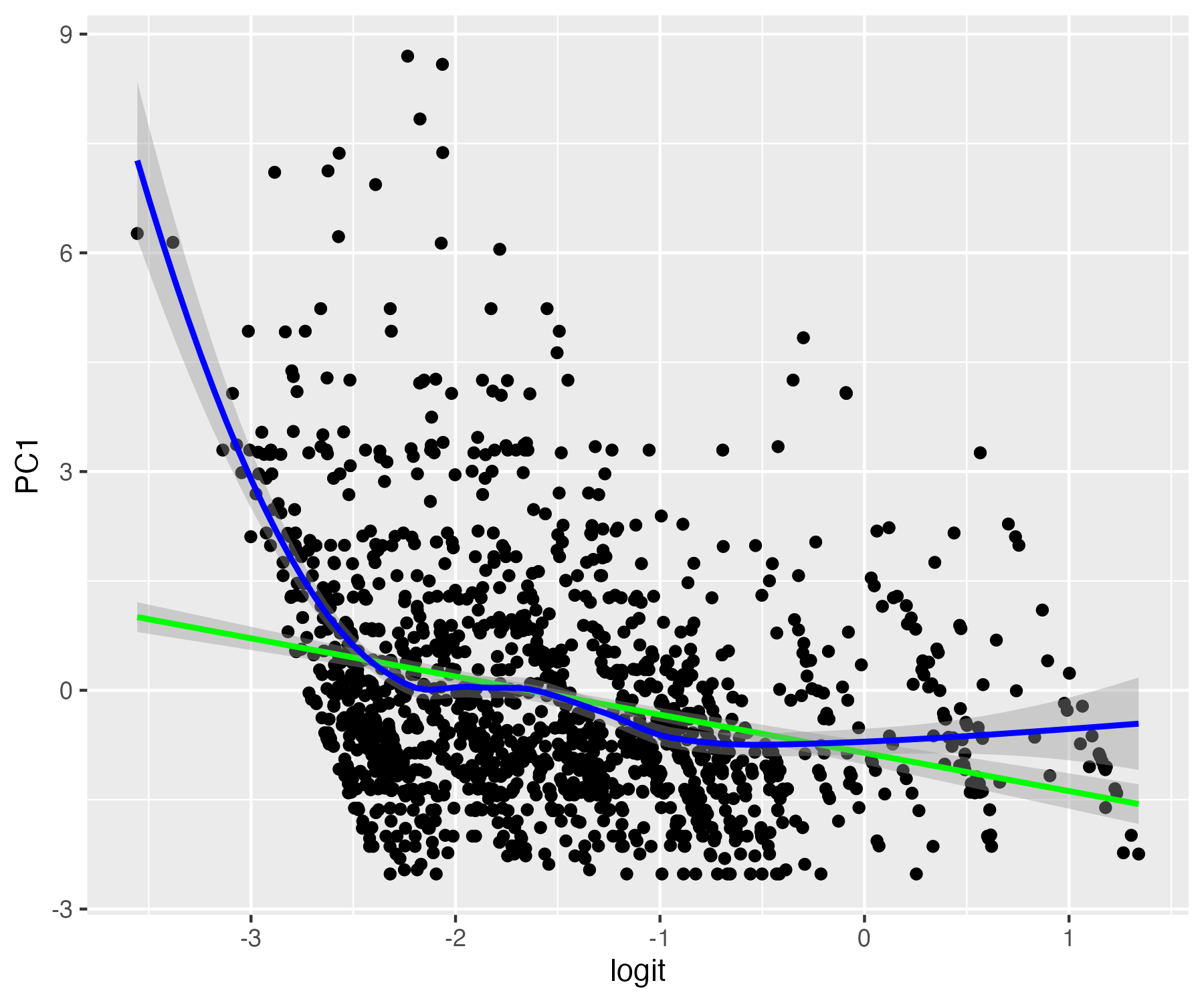


*Nostalgia*


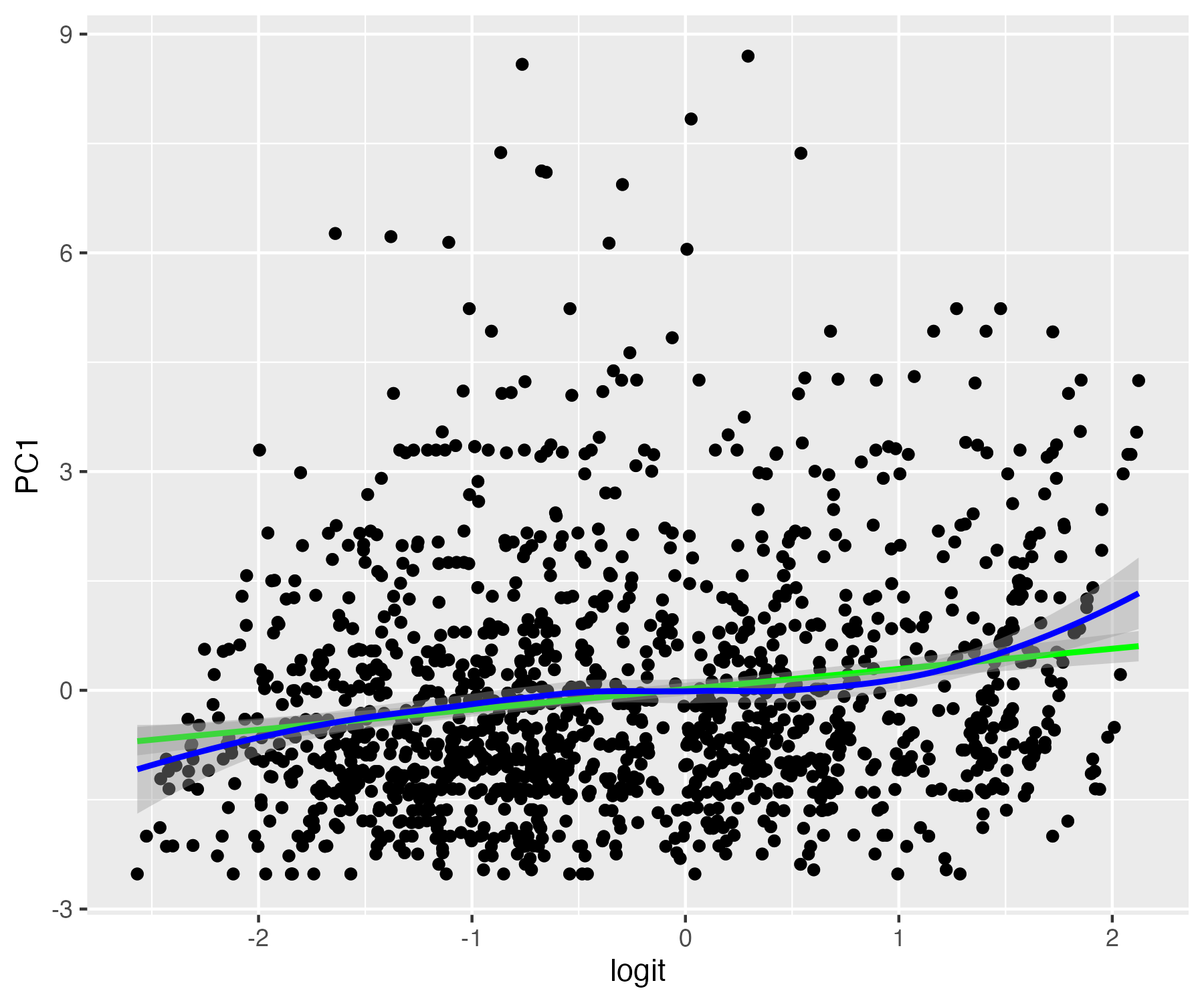


*Satisfaction*


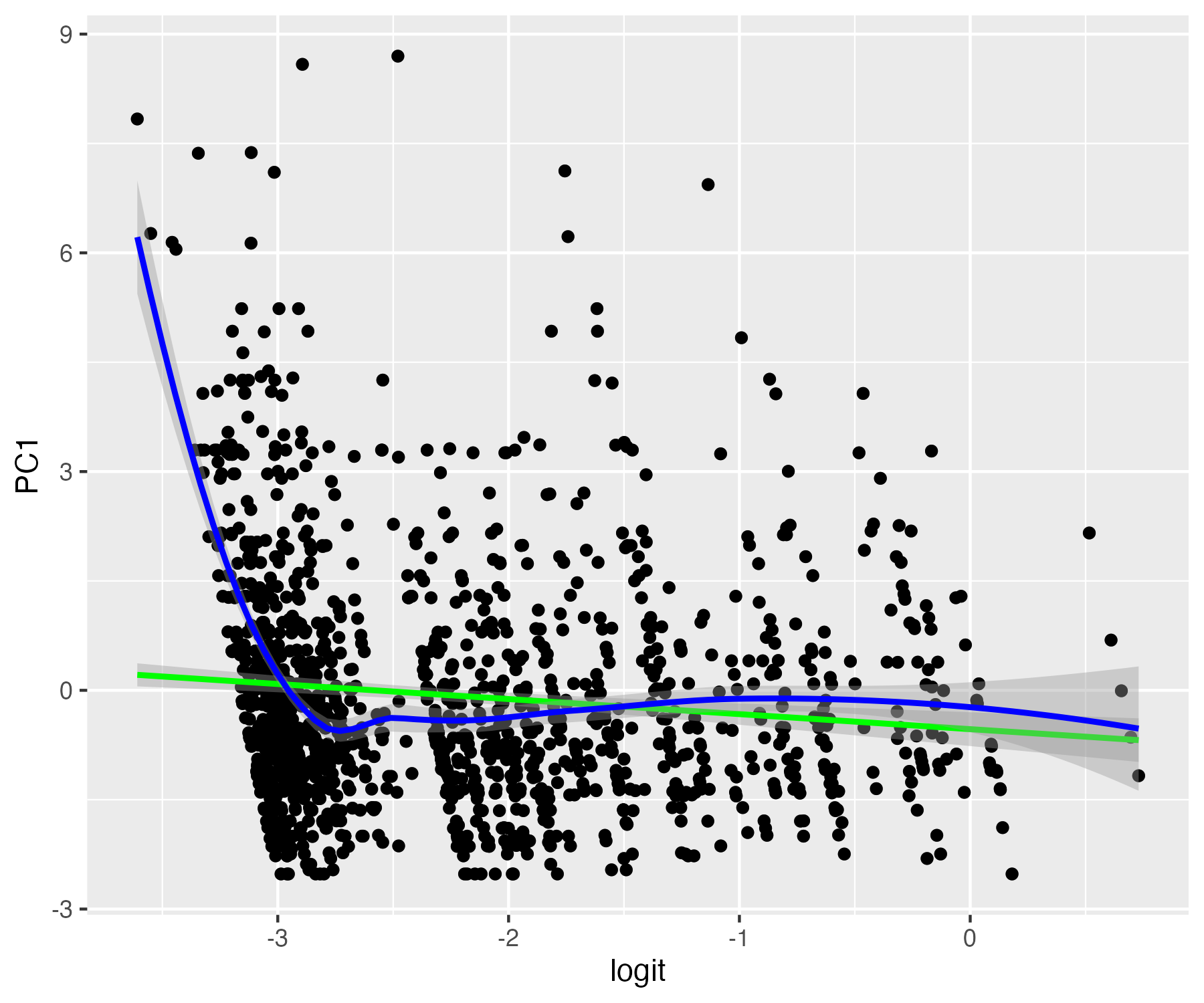


*Romance*


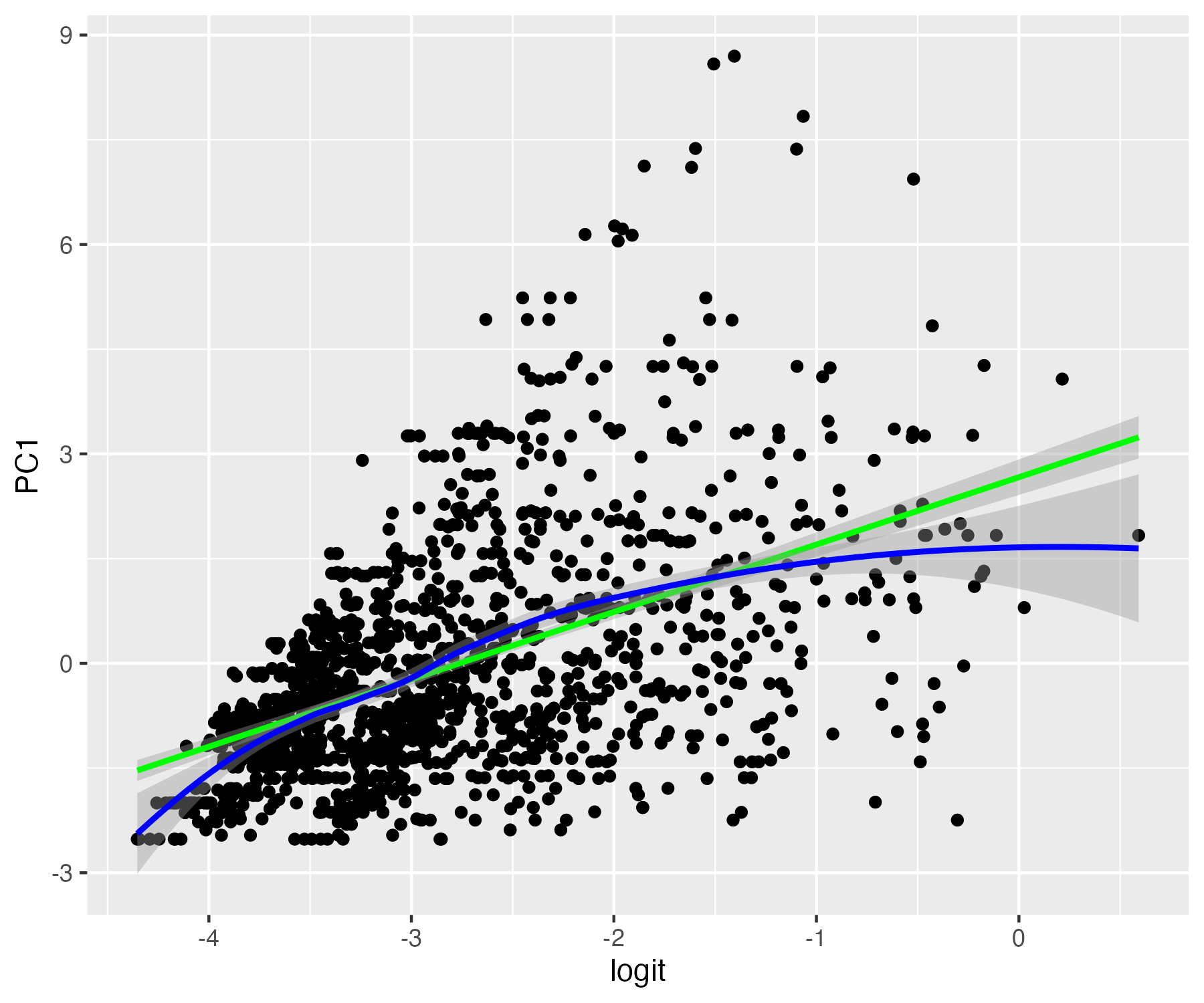


*Awe*


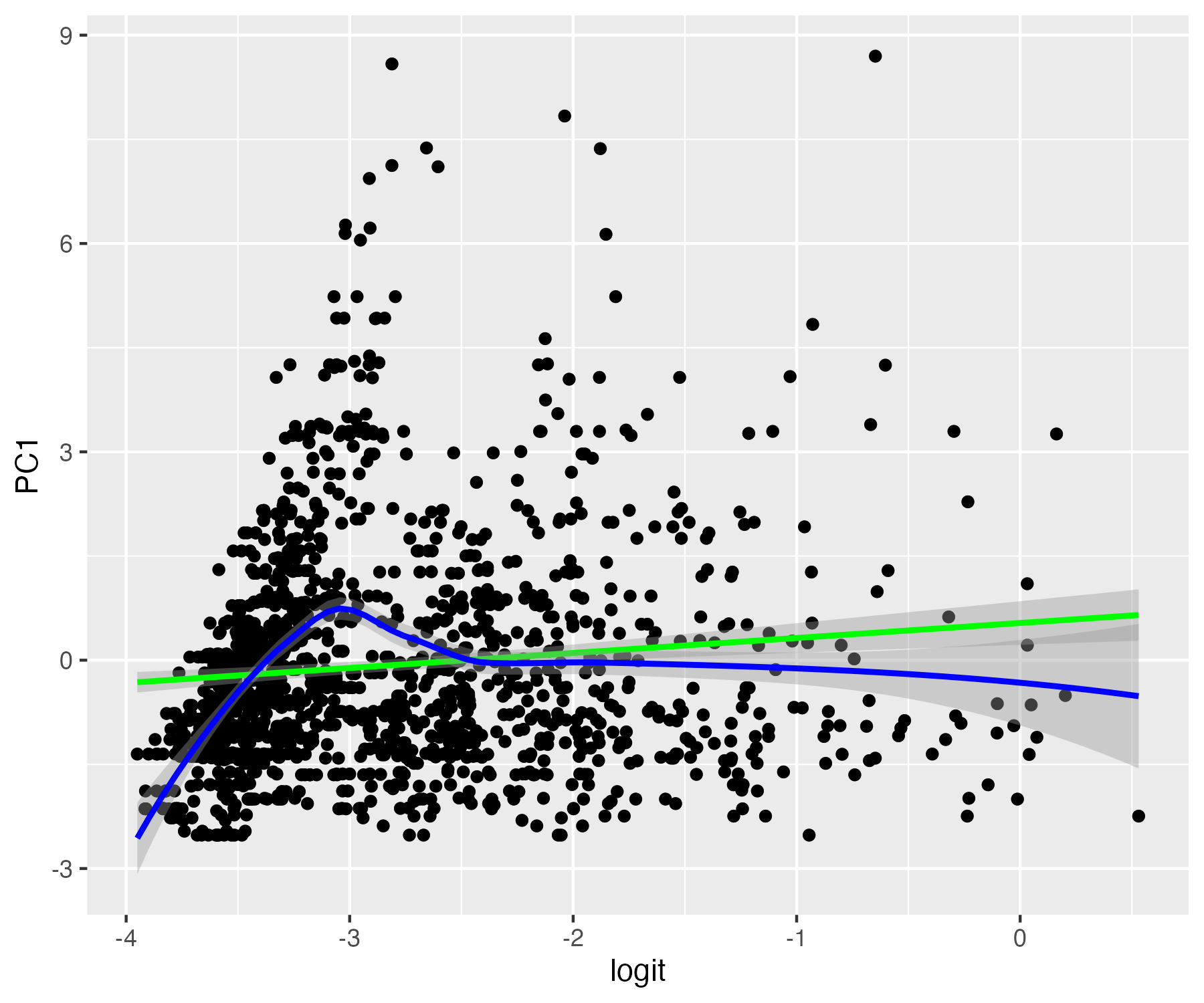


*Sadness*


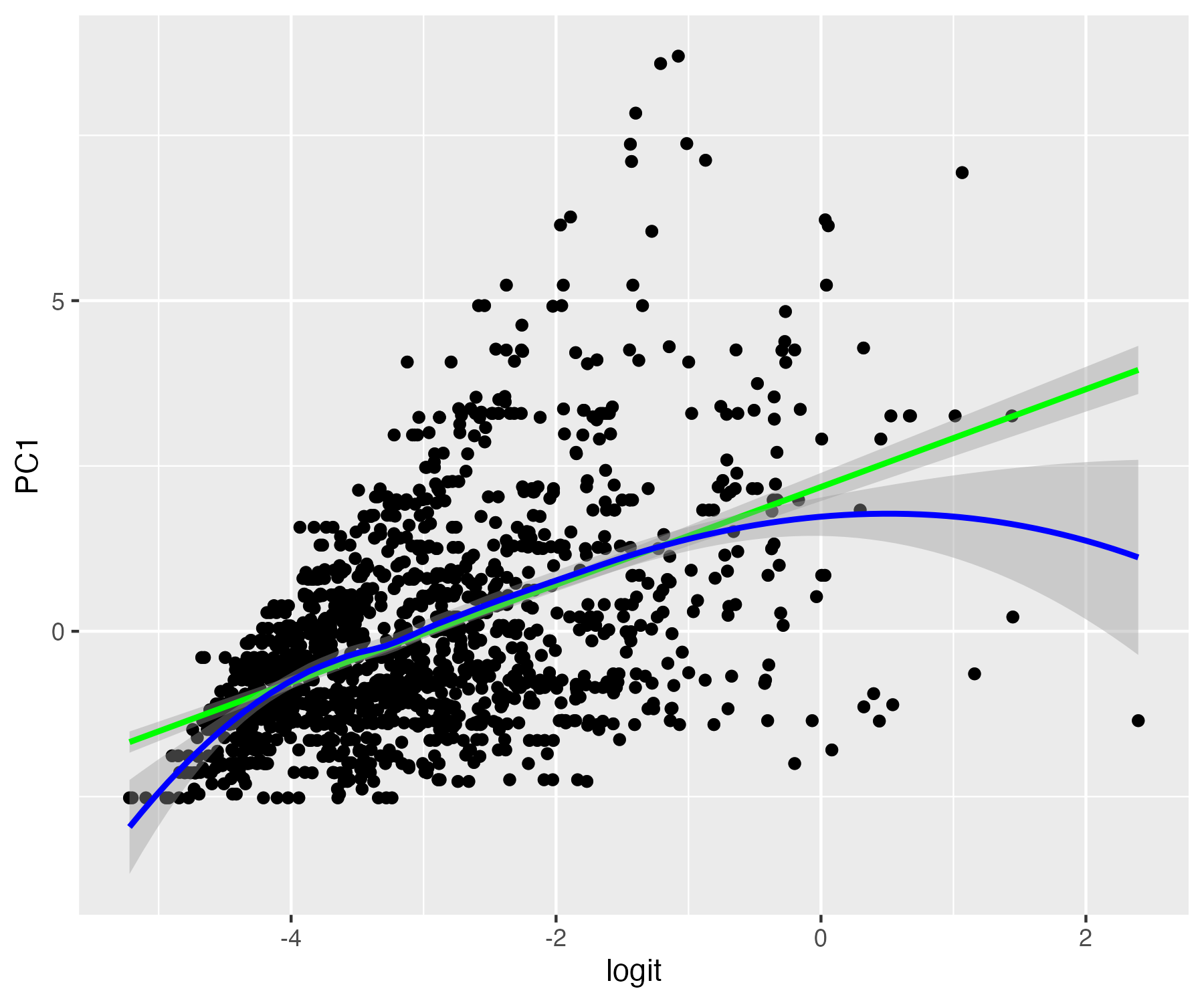


*Awkwardness*


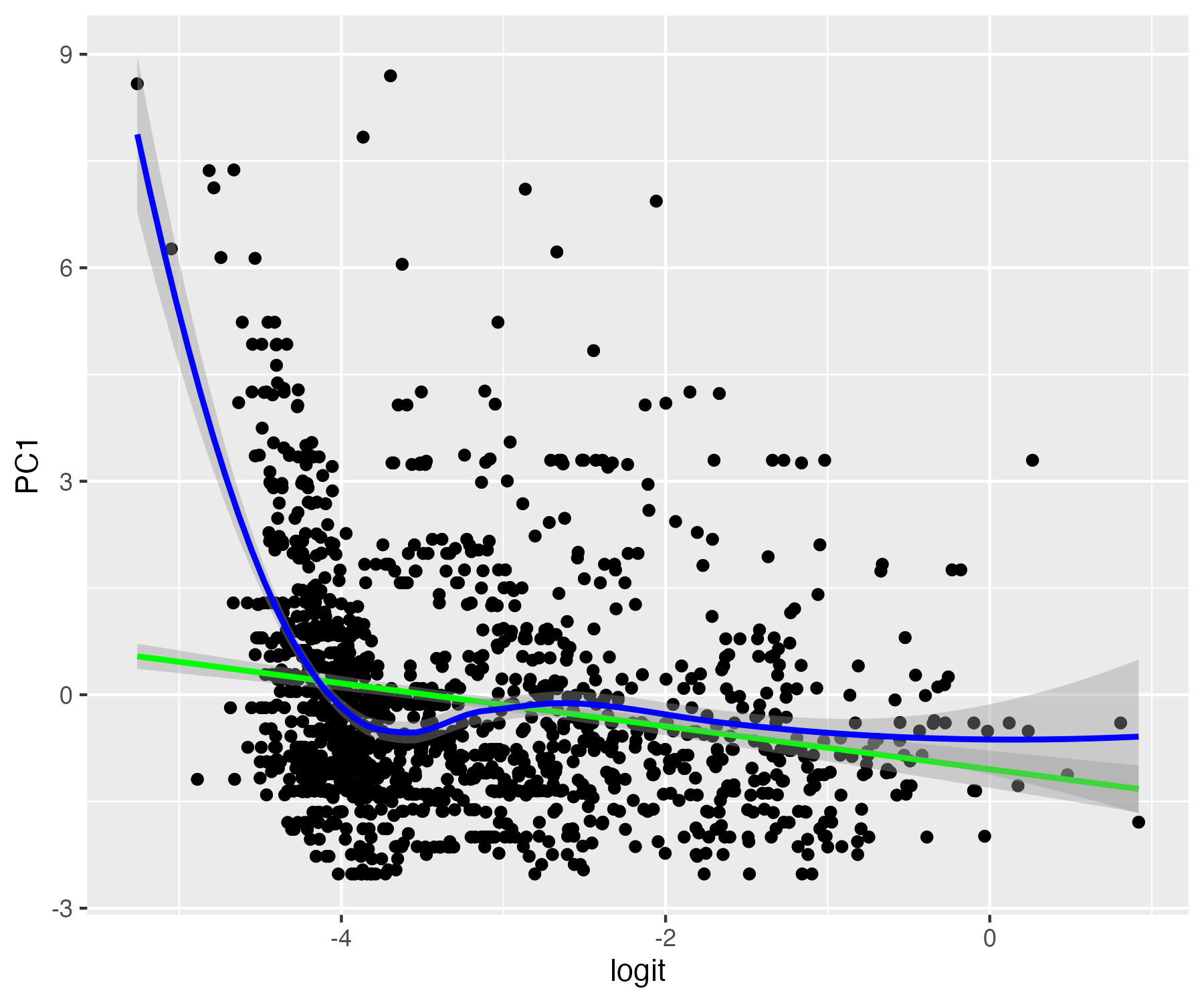

Supplement: S1 Appendix — In this supplementary appendix we report our complete data cleaning procedures and assumption testing for statistical models. (DOCX) [file pone.0329072.s001.docx]
